# A *Klebsiella pneumoniae* ST307 outbreak clone from Germany demonstrates features of extensive drug resistance, hypermucoviscosity, and enhanced iron acquisition

**DOI:** 10.1186/s13073-020-00814-6

**Published:** 2020-12-09

**Authors:** Stefan E. Heiden, Nils-Olaf Hübner, Jürgen A. Bohnert, Claus-Dieter Heidecke, Axel Kramer, Veronika Balau, Wolfgang Gierer, Stephan Schaefer, Tim Eckmanns, Sören Gatermann, Elias Eger, Sebastian Guenther, Karsten Becker, Katharina Schaufler

**Affiliations:** 1 Institute of Pharmacy, Pharmaceutical Microbiology, University of Greifswald, Friedrich-Ludwig-Jahn-Str. 17, 17489 Greifswald, Germany; 2 Central Unit for Infection Prevention and Control, University Medicine Greifswald, Greifswald, Germany; 3 Friedrich Loeffler-Institute of Medical Microbiology, University Medicine Greifswald, Greifswald, Germany; 4 Department of General, Visceral, Thoracic and Vascular Surgery, University Medicine Greifswald, Greifswald, Germany; 5 Institute for Hygiene and Environmental Medicine, University Medicine Greifswald, Greifswald, Germany; 6 IMD Laboratory Greifswald, Institute of Medical Diagnostics, Greifswald, Germany; 7 MVZ Laboratory Limbach Vorpommern-Rügen, Stralsund, Germany; 8 Department for Infectious Disease Epidemiology, Robert Koch-Institute, Berlin, Germany; 9 National Reference Centre for Multidrug-Resistant Gram-Negative Bacteria, Ruhr University Bochum, Bochum, Germany; 10 Institute of Pharmacy, Pharmaceutical Biology, University of Greifswald, Greifswald, Germany

**Keywords:** XDR *Klebsiella pneumoniae*, Outbreak, Hypervirulence, Plasmid transmission, “Mosaic” plasmid

## Abstract

**Background:**

Antibiotic-resistant *Klebsiella pneumoniae* are a major cause of hospital- and community-acquired infections, including sepsis, liver abscess, and pneumonia, driven mainly by the emergence of successful high-risk clonal lineages. The *K. pneumoniae* sequence type (ST) 307 lineage has appeared in several different parts of the world after first being described in Europe in 2008. From June to October 2019, we recorded an outbreak of an extensively drug-resistant ST307 lineage in four medical facilities in north-eastern Germany.

**Methods:**

Here, we investigated these isolates and those from subsequent cases in the same facilities. We performed whole-genome sequencing to study phylogenetics, microevolution, and plasmid transmission, as well as phenotypic experiments including growth curves, hypermucoviscosity, siderophore secretion, biofilm formation, desiccation resilience, serum survival, and heavy metal resistance for an in-depth characterization of this outbreak clone.

**Results:**

Phylogenetics suggest a homogenous phylogram with several sub-clades containing either isolates from only one patient or isolates originating from different patients, suggesting inter-patient transmission. We identified three large resistance plasmids, carrying either NDM-1, CTX-M-15, or OXA-48, which *K. pneumoniae* ST307 likely donated to other *K. pneumoniae* isolates of different STs and even other bacterial species (e.g., *Enterobacter cloacae*) within the clinical settings. Several chromosomally and plasmid-encoded, hypervirulence-associated virulence factors (e.g., yersiniabactin, metabolite transporter, aerobactin, and heavy metal resistance genes) were identified in addition. While growth, biofilm formation, desiccation resilience, serum survival, and heavy metal resistance were comparable to several control strains, results from siderophore secretion and hypermucoviscosity experiments revealed superiority of the ST307 clone, similar to an archetypical, hypervirulent *K. pneumoniae* strain (hvKP1).

**Conclusions:**

The combination of extensive drug resistance and virulence, partly conferred through a “mosaic” plasmid carrying both antibiotic resistance and hypervirulence-associated features, demonstrates serious public health implications.

**Supplementary Information:**

The online version contains supplementary material available at 10.1186/s13073-020-00814-6.

## Background

*Klebsiella pneumoniae* cause severe infections including sepsis, liver abscess, and pneumonia [1, 2]. The emergence of multidrug-resistant (MDR) and extensively drug-resistant (XDR), “classic” *K. pneumoniae* (cKp) has been mainly driven by the dissemination of high-risk clonal lineages and now constitutes a major global public health problem [3]. The majority of cKp strains cause infection in immunocompromised patients in healthcare settings and have demonstrated the ability to acquire antibiotic resistance elements. In addition to this cKp, a second pathotype termed hypervirulent *K. pneumoniae* (hvKp) is currently circulating, particularly in Asia but with increasing reports from other countries [2, 4]. The hvKp’s defining features are clinical characteristics including multiple site infection and metastatic spread in the healthy community [5] and, originally, a positive laboratory string test indicating a hypermucoid phenotype [6]. The definition of hypervirulence is controversial, however [7]. A recent study identified potential biomarkers including *peg-344*, *iroB*, *iucA*, plasmid-borne *rmpA* and *rmpA2* genes, and high siderophore production in hvKp to accurately differentiate the two pathotypes [8].

While *K. pneumoniae* sequence type (ST) 258 has been recognized as antibiotic-resistant, high-risk clonal lineage [9], ST307 came into spotlight only more recently [3, 10, 11]. The first *K. pneumoniae* ST307 isolates were obtained in the Netherlands in 2008 and Pakistan in 2009, followed by a period of sporadic isolations across Europe, Asia, Africa, and the Americas [11], and originated mostly, but not uniquely, from clinical samples [3, 10]. A phylogenetic study suggests the emergence of two deep-branching ST307 lineages, one of global relevance with genomes from locations worldwide and evidence of intercontinental transfer [10]. The other lineage included *K. pneumoniae* ST307 isolates from Texas, USA [12]. Within the global lineage, some isolates also originated from the infections in Texas [12], which suggests that the USA may have been the origin of this sequence type, especially as most of its genetic diversity was present in that location [10]. ST307 often carries transferable resistance-conferring genes against carbapenems and newer-generation cephalosporins like *bla*_KPC-3_, *bla*_NDM-1_, *bla*_OXA-48_, and *bla*_CTX-M-15_ [3, 10]. Resistance to the novel combination ceftazidime/avibactam [13] and to colistin was also reported [14, 15]. Besides that, the ST307 *K. pneumoniae* lineage comprises a variety of additional resistance and virulence determinants, integrative conjugative elements, and phages [3, 10]. There are several outbreak reports on MDR *K. pneumoniae* ST307 in clinical settings [16–18].

In this study, we analyzed carbapenemase-producing ST307 isolates, which have been recovered from screening and clinical samples within the course of an outbreak [19] that took place in four medical facilities in Western Pomerania, Germany, from June 2019 to October 2019. Additional cases were detected after the actual outbreak in the beginning of 2020. These isolates were characterized as carrying NDM-1 and OXA-48 carbapenemase-encoding genes, mostly simultaneously, and tested colistin-resistant. We performed whole-genome sequencing and phenotypic experiments to enable functional genomics for the in-depth understanding of this XDR outbreak clonal lineage.

## Methods

### Sequenced isolates and metadata

Between June 2019 and February 2020, we investigated 56 enterobacterial isolates from 25 different patients involved in the outbreak. In addition to *K. pneumoniae*, we included all other *Enterobacteriaceae* that matched the carbapenem-resistant phenotype. Most isolates were obtained from rectal (*n* = 23) or throat/tracheal secretion (*n* = 17) swabs collected as part of an extensive surveillance program in the affected clinical institutions (Additional file 2: Table S1). Initial antibiotic susceptibility testing (AST) was performed using the VITEK 2 (bioMérieux, Nürtingen, Germany) system and 96-well plate broth microdilution (Merlin, Bornheim-Hersel, Germany). Bacterial species were initially identified by MALDI-TOF MS (VITEK MS, bioMérieux, Nürtingen, Germany). For rapid detection of carbapenemase genes, a loop-mediated isothermal amplification (LAMP) assay (eazyplex SuperBug CRE, AmplexDiagnostics, Gars, Germany) was subsequently included in the University Medicine laboratory diagnostic program [19]. Almost 59% (33/56) of the isolates were assigned to “infection” samples. Clinical data and outcomes of 17 initial cases have been published elsewhere [19]. Most of these had severe underlying diseases. Patients were treated with ceftazidime-avibactam/aztreonam with synergistic activity. By October, six patients had died. Causal associations with the outbreak clone as well as clinical outcomes of other cases are still under investigation, however. Epidemiologic links among the different institutions were identified (Additional file 2: Table S1). Samples were incubated overnight on CHROMID CARBA and CHROMID ESBL agar plates (bioMérieux, Nürtingen, Germany), and single colonies were picked for identification with VITEK MS (bioMérieux, Nürtingen, Germany). Antimicrobial susceptibility testing was carried out using VITEK 2 (bioMérieux, Nürtingen, Germany), and in addition, 96-well plate broth microdilution was performed for determination of colistin MICs (Merlin, Bornheim-Hersel, Germany).

### Whole-genome sequencing

We generated 52 *K. pneumoniae*, 2 *E. coli*, 1 *C. freundii*, and 1 *E. cloacae* whole-genome sequences on different sequencing machines (MiGS: Illumina NextSeq 550; LGC: Illumina NextSeq 500; Eurofins: Illumina NovaSeq 6000). Two *K. pneumoniae* isolates (PBIO1953 [va20750], PBIO1951 [va19352]) were long-read sequenced using ONT’s Nanopore system. Additional file 2: Table S1 shows the respective metadata including origin institution, sampling location, and patient pseudonym. From 13 patients, more than one isolate was sequenced (11 patients with more than one *K. pneumoniae* isolate; 9 patients with more than one *K. pneumoniae* ST307 isolate). Some ST307 isolates were obtained from multiple sites from the same patient (e.g., PT17). DNA was extracted using the MasterPure™ DNA Purification Kit for Blood, Version II (Lucigen, Middleton, USA). After quantification and initial quality control, DNA was shipped to MiGS (Microbial Genome Sequencing Center, Pittsburgh, USA), LGC (LGC Genomics GmbH, Berlin, Germany), and Eurofins (Eurofins Genomics Europe Sequencing GmbH, Constance, Germany) and following library preparation with [20] (MiGS), the SeqWell™ Kit (LGC), and an adapted NEBNext Ultra™ II FS DNA Library Prep Kit (Eurofins) sequenced using 2 × 150 bp paired-end reads.

### Assembly and annotation

Raw sequencing reads were adapter-trimmed, contaminant-filtered, and quality-trimmed using BBDuk from BBTools v. 38.41 (http://sourceforge.net/projects/bbmap/). Both trimmed reads and raw reads were quality-controlled using FastQC v. 0.11.8 (http://www.bioinformatics.babraham.ac.uk/projects/fastqc/). De novo genome assemblies were conducted by employing the assembly pipeline shovill v. 1.0.4 (https://github.com/tseemann/shovill) in combination with SPAdes v. 3.13.1/3.14.0 [21]. As part of the pipeline, trimmed reads were subsampled to assemble at a maximum coverage of 100×. Besides the polishing step as part of the shovill pipeline, assemblies underwent an additional polishing step. For this, all trimmed reads were mapped back to the contigs using bwa v. 0.7.17 [22]. The obtained SAM/BAM files were sorted with Samtools v. 1.9 [23] and optical duplicates marked with GATK v. 4.1.2.0 [24]. Finally, variants were called with Pilon v. 1.23 [25]. The genomes of strains for which additional long-read sequencing data were obtained were hybrid-assembled with Unicycler v. 0.4.8 [26]. To verify the “hybrid” nature of plasmid 1, we mapped the long-reads of PBIO1953 back to the assembly using minimap2 v. 2.17 [27] and visualized the alignment with Tablet v. 1.19.09.03 [28] (Additional file 1: Fig. S1). The assembly graphs of putative plasmid recipient isolates and the closed reference isolate PBIO1953 were inspected with Bandage v. 0.8.1 [29] and its integrated BLAST hit (Megablast, ≥ 99% identity, *E* value 1e−10) visualization (Additional file 1: Fig. S2). Genome quality and completeness were assessed with CheckM v. 1.0.13 [30]. We used Prokka v. 1.14.1 [31] to annotate draft and finished genomes automatically.

### Genomic analysis

The in silico multi-locus sequence typing (MLST) and antibiotic resistance/virulence gene detection were carried out using mlst v. 2.19.0 (https://github.com/tseemann/mlst) and ABRicate v. 0.9.9 (https://github.com/tseemann/abricate), respectively. Both tools rely on 3rd-party public databases (e.g., PubMLST [32], VFDB [33], ResFinder [34], PlasmidFinder [35], BacMet [36]). To visualize draft genome content, BRIG v. 0.95-dev.0004 [37] and NCBI BLAST v. 2.9.0+ [38] with a threshold of at least 99% identity were employed by aligning isolate contigs against the respective reference. Pangenome analysis was performed with Roary v. 3.12.0 [39]. For in-depth typing of yersiniabactin, aerobactin, K locus, and O locus, we used Kleborate v. 1.0.0 with Kaptive [40–43]. A synteny plot comparing plasmid 1 (pPBIO1953_NDM-1) and plasmid 2 (pPBIO1953_CTX-M-15) of PBIO1953 with *K. pneumoniae* virulence plasmids pK2044 [44] and pKCTC2242 [45] was created with genoPlotR v. 0.8.9 [46].

### Phylogeny

**Fig. 1:**
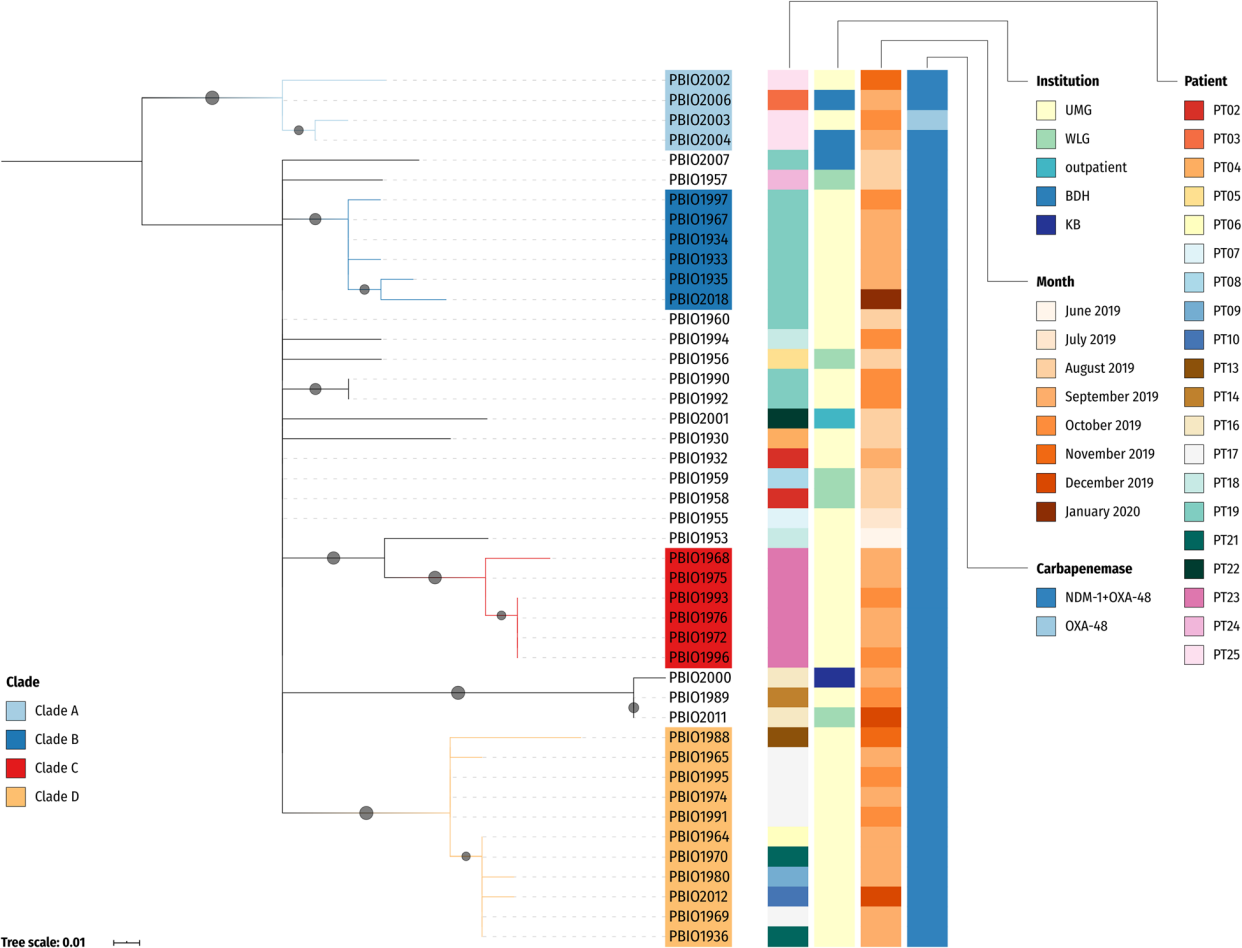
Core SNP phylogeny of *K. pneumoniae* ST307 isolates. The phylogenetic tree was inferred with a maximum likelihood-based approach and is based on a core SNP alignment (83 sites). The (midpoint-rooted) tree is shown with bootstrap proportions for values ≥ 50% (1000 replicates; filled circles on branches) and additional metadata. The patient associated with isolate PBIO1964 (PT06) was initially present on a submission ward for bacterial isolation but later on transferred to a different ward. Abbreviations: UMG, University Medicine Greifswald; WLG, clinic in Wolgast; BDH, clinic in Greifswald; KB, clinic in Karlsburg

We created a core SNP phylogeny for ST307 including (a) only the 44 ST307 isolates from this study (outbreak phylogeny) (Fig. 1) and (b) the 44 ST307 isolates as well as 97 publicly available ST307 genomes (79 as raw reads, 18 as assembly) (global phylogeny) (Additional file 2: Table S2). SNPs were called from the finished genome of PBIO1953 using snippy v. 4.4.1 (https://github.com/tseemann/snippy). Alignments were filtered for recombinations using Gubbins v. 2.4.1 [47] and core SNPs extracted using snp-sites v. 2.5.1 (83 sites for the outbreak phylogeny; 1476 sites for the global phylogeny) [48]. A maximum likelihood tree was inferred with RAxML-NG v. 0.9.0 [49] using GTR+G. The best-scoring maximum likelihood tree was midpoint-rooted in iTOL v. 5.5 [50] and visualized with metadata. An additional phylogenetic tree of all isolates was inferred based on whole genomes with the help of JolyTree v. 1.1b.191021ac [51] and visualized with iTOL v. 5.5 [50].

### Pathway analysis of variants

Single-nucleotide polymorphisms detected among the *K. pneumoniae* ST307 genomes were filtered for missense, frameshift, and stop-gained variants and respective genes subjected to metabolic pathway analysis using the EcoCyc (https://ecocyc.org/) [52] database (reference: *Klebsiella pneumoniae* “KpvST383_NDM_OXA-48” [NCBI Biosample number: SAMN10409888] [53]).

### Bacterial isolates and PCV construction

For phenotypic experiments, we used a selection of five *K. pneumoniae* ST307 outbreak isolates (PBIO1953, PBIO2000, PBIO1935, PBIO1994, PBIO1993) and three non-ST307 *K. pneumoniae* present during the outbreak (PBIO1951 and PBIO1961 [ST395], PBIO1979 [ST405]) (Additional file 2: Table S1). They were compared against three *K. pneumoniae* with different sequence types: a common ESBL-producing reference (ATCC700603 [ST498]), one of the ubiquitously occurring *K. pneumoniae* lineage ST15 (PBIO2008), and one archetypal, hypervirulent *K. pneumoniae* strain (hvKP1, ST86) [6, 54]. In addition, we included a non-carbapenemase-producing *K. pneumoniae* ST307 isolate from a previous study (IMT38405 [PBIO2009]) [11] and a “plasmid-cured” variant (PCV1935) stemming from PBIO1935. Based on a previously published protocol [55], this mutant was constructed by culturing PBIO1935 for 7 days at 42 °C and using ceftazidime/avibactam (16 μg/mL) as selection marker to identify NDM-1-plasmid-cured variants. Plasmid profile analysis (Additional file 1: Fig. S3A) performed as previously described [55], and bioinformatics analysis confirmed partial loss of plasmid 1 (Additional file 1: Fig. S3A; dashed box) and complete loss of plasmid 3 (Additional file 1: Fig. S3A; dotted box) in PCV1935.

### Growth curves

Growth curves in LB medium were performed using standardized protocols. Experiments were performed using three technical replicates and three biological replicates [56]. An *E. coli* K-12 strain (W3110) was used as control. Growth rates were calculated as follows: μ = (ln(CFU/mL *t*_1_) − ln(CFU/mL *t*_0_))/*t*_1_ − *t*_0_.

### Hypermucoviscosity

Hypermucoviscosity experiments were performed using the string test. Strings of 5 mm or longer that formed after stretching on the tip of a sterile inoculation loop were defined positive [6]. Experiments were performed with three technical replicates and three biological replicates.

### Siderophore secretion

We analyzed the study’s set of isolates for their ability to secrete siderophores using an adapted method described by Schwyn and Neilands [57]. Fifty microliters of overnight cultures of the isolates was grown in 5 mL fresh LB medium to an OD_600_ of 0.6. Five microliters of this culture was put on agar plates containing chrome azurol S-iron(III)-hexadecyltrimethylammonium bromide and incubated overnight at 37 °C. Iron uptake was determined visually by color shift from blue to yellow the following day. One ST131 *E. coli* strain (IMT21183) and one K-12 *E. coli* strain (W3110) were included as controls [58]. Experiments were performed with three technical replicates and three biological replicates.

### Biofilm formation

We used the same set of isolates as described above and performed a biofilm formation experiment as described in previous reports [59]. Ten microliters of overnight cultures of all isolates was transferred to 1 mL LB medium and cultured at 37 °C until OD_600_ values of 0.6–0.8 were reached. One hundred microliters of culture per well was transferred to a 96-well microtiter plate. After 3 days of static culture at 37 °C, planktonic cells were removed from the liquid medium. The wells were washed three times with 150 μL of double-distilled water, and the majority of the biofilms was stained with 150 μL of 0.1% crystal violet (CV) for 30 min. Then, the unbound dye was removed, and the plates were again washed. Finally, the CV binding to the biofilm was dissolved in 150 μL of 95% ethanol for 30 min, and biofilm formation was quantified by measuring the absorbance at OD_590_ with a microplate reader (Fluostar Omega, BMG Labtech, Ortenberg, Germany). Experiments were performed with three technical replicates on individual 96-well microtiter plates and three biological replicates. One biofilm-negative (PBIO729) and one positive control (W3110) [56] were included.

### Serum resistance

We performed serum resistance experiments in human serum (Pan-Biotech GmbH, Germany) as described previously [60]. We inoculated 5 μL of overnight culture in 495 μL fresh LB medium and incubated at 37 °C for 1.5 h. Inoculum was centrifuged for 3 min and resuspended in 1 mL of sterile 1× PBS. Thirty microliters was added in triplicates to 96-well microtiter plates containing 270 μL of 50% human serum. Thirty microliters of the sample was collected from each well, serially diluted, plated on LB plates, grown at 37 °C overnight, and counted the next day (0-h count). The 96-well microtiter plates were incubated for 4 h at 37 °C. Following incubation, 30 μL of the culture was plated, incubated, and again counted (4-h count). Growth in serum was obtained by determining differences in the CFU after 4 h of incubation compared to the 0-h count. Experiments were performed with three technical replicates on individual 96-well plates and three biological replicates. An *E. coli* serum-resistant control (PBIO1289, ST1159 [61]) was included.

### Desiccation tolerance

Desiccation tolerance experiments for the study’s set of isolates were performed as described previously with some modifications [59]. A single colony was cultured in 10 mL liquid LB broth until bacterial cells reached an OD_600_ value of 0.6–0.8. One hundred microliters was serially diluted, plated on LB plates, grown at 37 °C overnight, and counted the next day (C0d, 0-day count). Another 100 μL of the same culture was transferred into a 96-well microtiter plate. Subsequently, the plate was transferred into a sterile dryer with dehydrated silica gel. The dryer was placed in a sterile incubator (Mytron, Heilbad Heiligenstadt, Germany), which was kept at a constant temperature of 37 °C. After 6 days of drying, the 96-well microtiter plate was removed, 100 μL/well of fresh medium was added, and the plate was cultured with 200 rpm shaking at 37 °C for 3 h. One hundred microliters was collected from each well, serially diluted, plated on LB plates, grown at 37 °C overnight, and counted the next day (C6d, 6-day count). Experiments were performed with three technical replicates on individual 96-well microtiter plates and three biological replicates. An *E. coli* K-12 strain (W3110) was included as control.

### Heavy metal tolerance

Overnight cultures of the study’s set of isolates were adjusted to McFarland standard 0.5, and 50 μL of a 1:200 dilution of adjusted suspensions in Mueller-Hinton broth (Roth, Karlsruhe, Germany) was used as inoculum for incubations for 16 to 20 h at 37°C in heavy metal-containing microtiter plates (Merlin Biocide plates, Bornheim-Hersel, Germany). The plates contained a wide range of concentrations of three heavy metals: zinc chloride [4–8192 μg/mL], copper sulfate [32–8192 μg/mL], and silver nitrate [0.5–64 μg/mL]. We used a sealing tape to prevent dehydration of the plates. After incubation, the minimum inhibitory concentration was determined visually and reported as the tolerance breakpoint. Experiments were performed with three technical and three biological replicates. *E. coli* ATCC25922 was used as control isolate.

### Statistics

Statistics were performed using GraphPad Prism 8.0 (https://www.graphpad.com/). After investigating Gaussian distributions, the non-parametric Kruskal-Wallis test [62] was applied for multiple comparisons of bacterial groups using median values. Bonferroni adjustment was applied, which resulted in corrected *p* values of *p* < 0.016 to assess significant changes [63]. Pairwise comparison of growth rates (μ) between all *K. pneumoniae* ST307 isolates and PBIO1961 was performed using the Mann-Whitney *U* test (*p* < 0.05).

## Results

### Genomic analysis and phylogeny

The XDR *K. pneumoniae* outbreak clone was first detected at the University Medicine Greifswald on June 25, 2019 (Additional file 2: Table S1), following bacterial screening of a tracheal secretion sample [19]. Of 52 *K. pneumoniae*, 44 belonged to sequence type (ST) 307, three to ST395, three to ST11, and one each to ST405 and ST147. The two *E. coli* were ST405 and ST362, whereas *C. freundii* was a ST153 and *E. cloacae* a ST45 isolate (Additional file 2: Table S1). We focused mainly on the phylogenetics and phenotypes of the ST307 outbreak but included accessory and non-ST307 genomes to investigate transmission of resistance plasmids within the bacterial species and to others.

When comparing all 44 ST307 against the closed reference genome of PBIO1953, our analysis revealed 22 single-nucleotide polymorphisms (SNPs) at most (minimum, 6 [PBIO1958, PBIO1960, PBIO1932]; maximum, 22 [PBIO2004]; median, 11). As expected, the phylogram shows a homogenous picture (Fig. 1; Additional file 1: Figure S4) with several sub-clades. There are sub-clades that include only isolates originating from the same patient (clades B [8–11 SNPs compared to PBIO1953] and C [6–8 SNPs compared to PBIO1953]) and sub-clades that comprise isolates from different patients (clades A [14–17 SNPs compared to PBIO1953] and D [11–15 SNPs compared to PBIO1953]) suggesting recent transmission between patients. Epidemiologic data support these results, for example for sub-clade D: PT17 with isolates PBIO1965, PBIO1969, PBIO1974, PBIO1991, PBIO1995, and PT21, from whom we obtained isolates PBIO1936 and PBIO1970, stayed on the same ward during the same time (September 2019). On the contrary, PT06 with isolate number PBIO1964 was present on a different ward during September 2019. Note, however, that PT06 was transferred later on. Interestingly, this patient underwent endoscopy examination with the same endoscope used for PT17 and PT21. It thus seems possible that *K. pneumoniae* ST307 was transmitted among patients either by cross-contamination through healthcare workers and surfaces or by an endoscope as has been described previously [64].

When we placed the outbreak phylogeny in a global context (Additional file 1: Fig. S4), we noticed that a cluster of KPC-producing ST307 genomes originally obtained from the United Kingdom (UK) (Additional file 2: Table S2) was the phylogenetically closest to our ST307 outbreak isolates.

We then explored the distribution and character of SNPs among the ST307 genomes (with PBIO1953 as reference) further (Additional file 1: Fig. S5). All polymorphisms that were not in coding sequences (CDS) or not assigned as missense, frameshift, or stop-gained variants were excluded from our subsequent analysis. We further excluded all insertion and/or deletion mutations (Indels). Variants accumulated almost uniquely in chromosomally encoded genes (59/66). When analyzing the data of 44 annotated genes after exclusion of hypothetical proteins in EcoCyc, we noticed that pathways related to membrane transport (14/44), regulation and signal transduction (9/44), amino acid and sugar metabolism (12/44), DNA-replication/conjugation (5/44), and lipopolysaccharide biosynthesis (4/44) were often affected by mutations. Thirty-seven genomes harbored a missense SNP in the conjugation gene *traI* (plasmid 3) and, simultaneously, the topoisomerase gene *gyrA*, which are both involved in plasmid conjugation and transfer [65, 66]. Interestingly, all potential *K. pneumoniae* ST307 plasmid donors belonged to this set of genomes.

We often found variants in genes encoding for membrane efflux. While in sub-clade C, *sotB* was affected, in other clusters, we observed missense mutations in *phoE*, *gltC*, and *ompC*. This might be an example for phenocopy in isolates of different sub-clades. The nitrate/nitrite sensor gene *narX*, differentiating sub-clades B (PT19) and D (various patients), and sensor protein *pmrB*, differentiating sub-clade C (PT23) from other sub-clades (various patients), were repeatedly and independently mutated among different patients. In addition, two other genes, *narI* and *narJ*, displayed SNPs; both are involved in the regulation of anaerobic respiratory gene expression in response to nitrate and nitrite.

Several genomes obtained from the same patient demonstrated identical variants not present in other genomes, which could be explained by either a disruption of the infection chain between patients or the non-advantageous character of the mutation for dissemination. One example is the variant in *btsT*, a gene involved in pyruvate uptake and present in two isolates from the same patient over a period of 7 days. Interestingly, the number of missense/nonsense SNPs did not significantly increase over time during the course of the outbreak compared to the earliest isolate PBIO1953 (for example PBIO1955, 8 SNPs; PBIO1956, 11 SNPs; PBIO1957, 11 SNPs; PBIO2011, 11 SNPs; PBIO2012, 13 SNPs; and PBIO2018, 12 SNPs; Additional file 1: Fig. S5, top).

All ST307, with the exception of PBIO2003, carried *bla*_NDM-1_, *bla*_OXA-48_, and *bla*_CTX-M-15_ resistance genes simultaneously, which was consistent with their phenotypes. Due to the fact that *mcr*-genes were not present, phenotypic resistance against colistin could not be explained by the expression of such. We identified several missense mutations in the two-component systems PhoP/PhoQ (*phoQ*: 89T>A [Leu30Gln]) and PmrA/PmrB (*pmrA*: 121G>A [Ala41Thr]; *pmrB*: 637C>A [Leu213Met], 766G>C [Gly256Arg]), typically involved in colistin resistance conferred by chromosomal point mutations [67]. Additional mutations were present in *eptA* (*pmrC*) (80T>G [Phe27Cys]), *pmrD* (179C>T [Thr60Met]), *arnT* (1115A>G [Lys372Arg], 1211C>G [Ser404Cys]), and *ugd* (1061A>C [Asp354Ala]) (Additional file 1: Fig. S6). Three mutations (*pmrB*: 766G>C; *eptA*: 80T>G; *arnT*: 1115A>G) were present in colistin-susceptible isolate PBIO1979 (ST405). Among the phenotypically colistin-resistant isolates, four carried additional mutations in *pmrA* (PBIO2001: 518 T>A [Ile173Asn], 533T>C [Ile178Thr]), *pmrB* (PBIO1953: 364G>C [Glu122Gln]), or *phoP* (PBIO1990, PBIO1992: 563A>C [His188Pro]). Interestingly, one missense mutation in *pmrB* (604C>A [Gln202Lys]) was exclusively present in isolates from patient PT23. While the amino acid substitutions in PmrA/PmrB (Ala41Thr/Leu213Met; Gly256Arg), together with an insertional inactivation of *mgrB*, were previously reported in a colistin-resistant *K. pneumoniae* ST307 isolate in 2015 [14], both this study’s colistin-susceptible and colistin-resistant isolates showed an uninterrupted *mgrB*, whose gene product is a small negative regulator of PhoQ. The colistin-resistance phenotype is possibly explained by the combination of several mutations in chromosomally encoded genes.

Our analysis revealed that all ST307 genomes were positive for the chromosomally encoded “yersiniabactin (*ybt*) lineage 10” genetic makeup, associated with the *K. pneumoniae* integrative conjugative element 4 (ICE*Kp4*). The corresponding yersiniabactin sequence type (YbST) was 20-2LV, which is most similar to YbST20 but varies in two loci (*irp2*: allele 61 instead of allele 208; *fyuA*: allele 39 instead of allele 2). All ST307 shared “aerobactin lineage *iuc* 1” signatures with aerobactin sequence type (AbST) 63-1LV and 63 (PBIO2003); thus, all ST307 isolates but one had a single-locus variant of AbST63 (SNP in *iutA*). Capsule (K) and O antigen loci of *K. pneumoniae* ST307 isolates were predicted as KL102 and O2 variant 2 (O2v2) with identities of ≥ 99.25% and ≥ 98.45%, respectively (Additional file 2: Table S1).

**Fig. 2:**
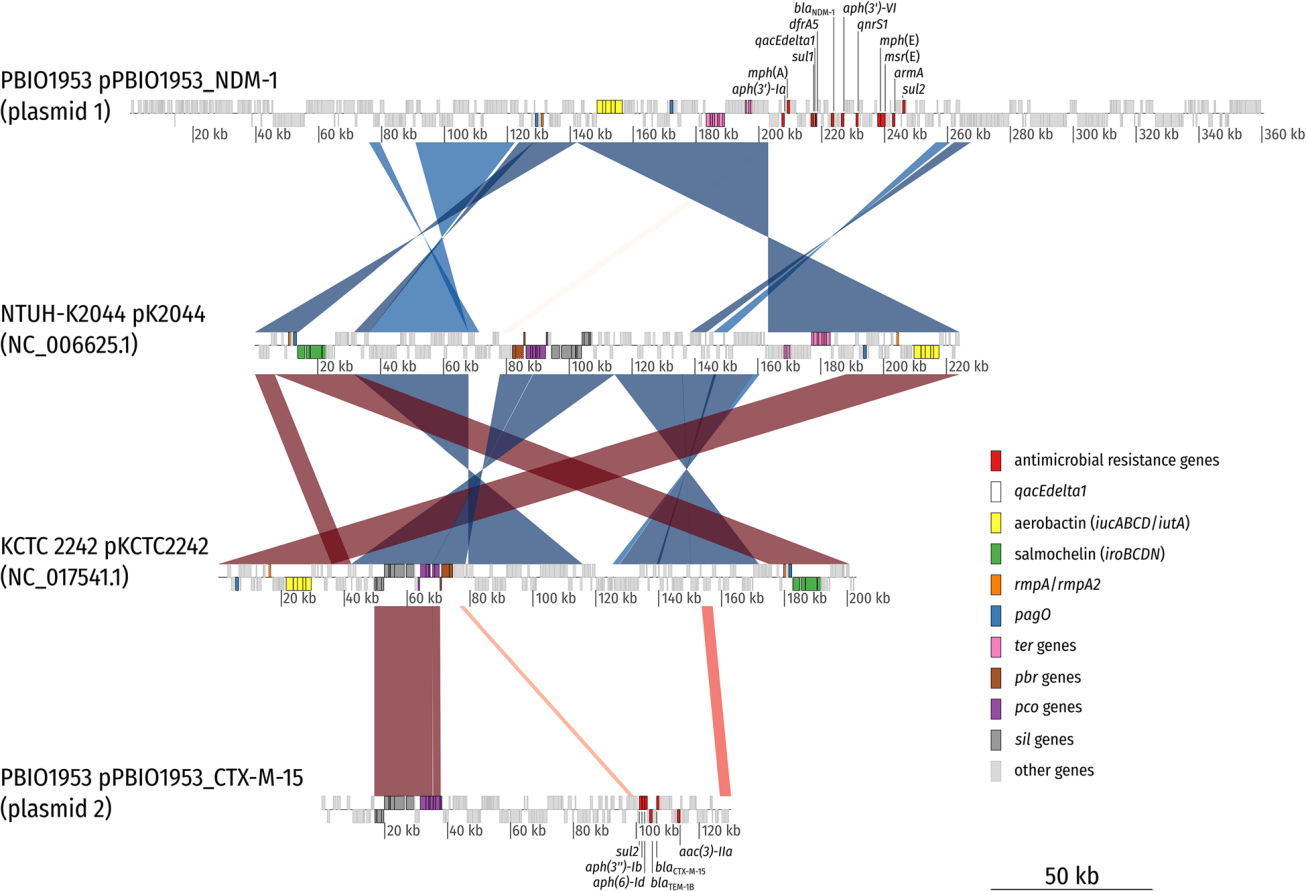
Synteny plot of hybrid resistance/virulence plasmid 1 (pPBIO1953_NDM-1) and resistance plasmid 2 (pPBIO1953_CTX-M-15). The plot depicts pairwise BlastN comparisons (*E* value 1e−10) between pPBIO1953 plasmid 1 and 2 and pK2044 and pKCTC2242. Alignment lengths were required to be at least 1% of the smaller replicon in the comparison to be included. Direct comparisons are colored with red hues whereas reverse comparisons are colored with blue hues. Boxes on top depict CDS on the forward strand, and those at the bottom depict CDS on the reverse strand. Boxes with a dotted outline are annotated as pseudo CDS. Plasmid 1 of PBIO1953 combines virulence features, such as aerobactin, with acquired antimicrobial resistance genes (e.g., *bla*_NDM-1_), which are missing from typical virulence plasmids of hypervirulent strains NTUH-K2044 and KCTC 2242. Note that PBIO1953 plasmid 2 carries several metal resistance genes (*pco* and *sil* genes) present on hvKP virulence plasmids but absent from plasmid 1

Interestingly, the NDM-1, CTX-M-15, and OXA-48 resistances were located on three different, large plasmids, based on their respective size subsequently termed plasmids 1 (pPBIO1953_NDM-1), 2 (pPBIO1953_CTX-M-15), and 4 (pPBIO1953_OXA-48) (Additional file 1: Fig. S3 and S7). Plasmid 1 (size = 360,596 bp; incompatibility [Inc] types: IncFIB, IncHI1B) did not only encode the New-Delhi metallo-beta-lactamase (NDM)-1 but also several other resistance genes (*dfrA5*, *sul1*, *sul2*, *qnrS1*, *aph (3′)-Ia*, *aph (3′)-VI*, *armA*, *mph*(A), *mph*(E), and *msr*(E)) as well as virulence (*peg-344/pagO*, *peg-1860/pagO* [metabolite transporter], *iucABCD*, *iutA* [aerobactin], and *rmpA/rmpA2* [regulator of mucoid phenotype]) and disinfectant/mineral resistance factors (*qacEdelta1* [disinfectant resistance], *ter* [tellurite resistance]) (Additional file 1: Fig. S7B). Plasmid 2 (size = 130,131 bp; IncFIB) carried the following antibiotic resistances: *bla*_CTX-M-15_, *bla*_TEM-1B_, *sul2*, *aac (3)-IIa*, *aph (3″)-Ib*, and *aph (6)-Id*, and, interestingly, several genes responsible for heavy metal resistance: *ars* (arsenic/antimony resistance), *sil* (silver resistance), and *pco* (copper resistance). We compared plasmids 1 and 2 to two well-characterized, typical virulence plasmids of hypervirulent *K. pneumoniae* NTUH-K2044 [44] and KCTC 2242 [45]. We found a high degree of similarity in the aforementioned virulence and mineral resistance features (Fig. 2). This is of particular public health interest as the emergence of “mosaic” plasmids carrying AMR and virulence factors can confer both enhanced virulence and multidrug resistance in one single transfer. Plasmid 4 (size = 63,589 bp; IncL/M) was positive for the OXA-48-encoding gene. Two additional plasmids with sizes of 72,679 bp (plasmid 3; IncFII) and 6656 bp (plasmid 5; Col440I) did not carry any resistance or virulence genes (Additional file 1: Fig. S7D and S7F).

**Fig. 3:**
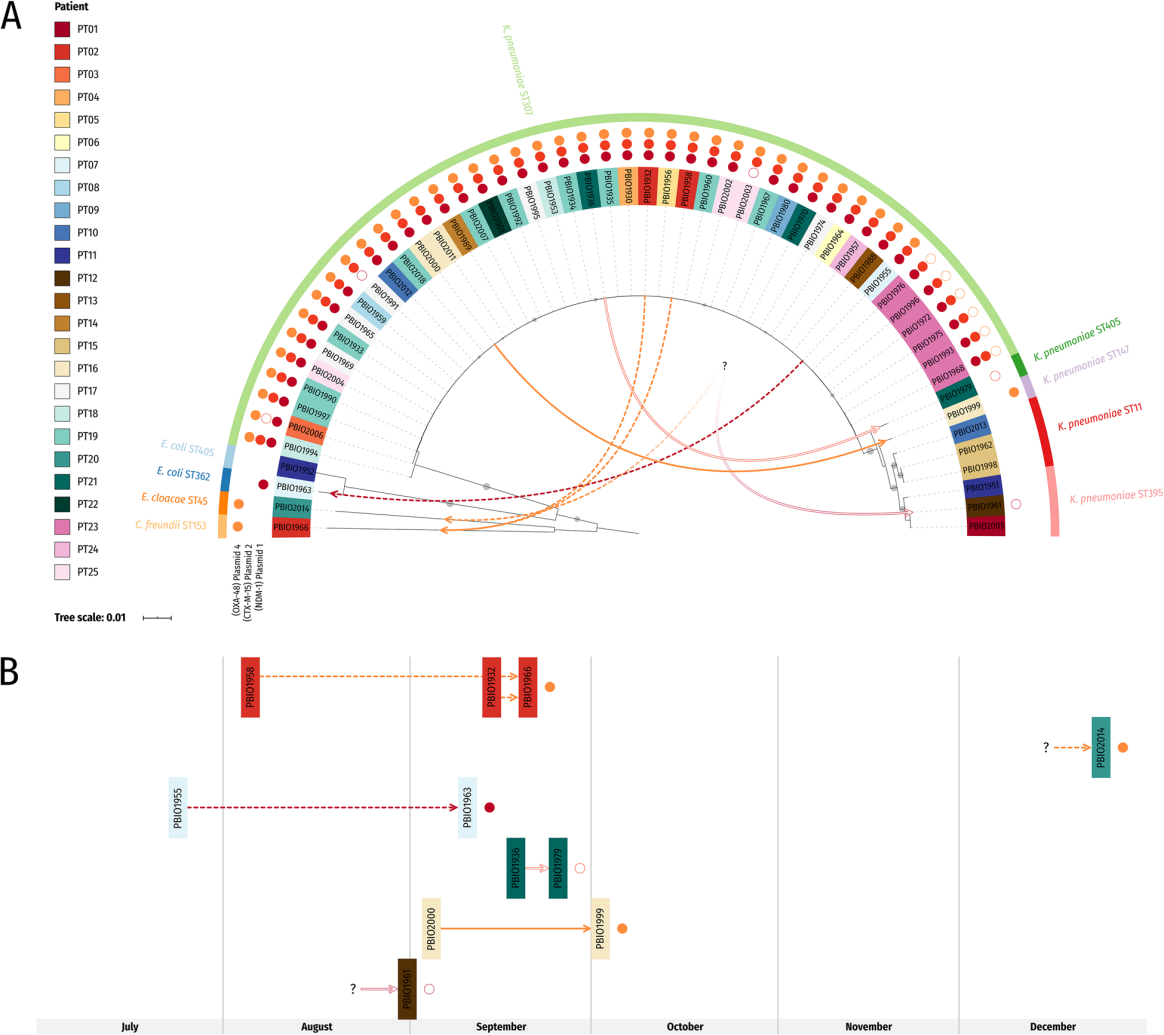
Putative plasmid transfer among *K. pneumoniae* isolates and between different bacterial genera. Solid arrows indicate putative intra-species transfer, whereas dashed arrows indicate inter-bacterial transfer. The putative inter-patient transfer is illustrated through a question mark. Note that from PT12 (PBIO1961), no additional carbapenem-resistant isolates were obtained and that no other ST307-positive patient was simultaneously present on the same ward whereas inter-patient transfer for PT20 (PBIO2014) is further supported by the epidemiologic data. Line colors match the colors of transferred virulence/resistance plasmids and are also shown next to the isolate names as circles. Closed circles—complete plasmid backbone (Megablast hits with identity ≥ 99%: coverage ≥ 99%). Open circles—incomplete plasmid backbone (Megablast hits with identity ≥ 99%: coverage > 77% and < 99% (Additional file 2: Table S4)). **a** The phylogenetic tree is based on distance-corrected MinHash dissimilarities between sequenced genomes and was inferred with FastME as part of JolyTree. Shown at the branches is the rate of elementary quartets (REQ) for values ≥ 0.75 (circle diameter). **b** Timeline figure of plasmid transfer between July and December 2019

When comparing these plasmid sequences to the other genomes (Fig. 3, Additional file 1: Fig. S2, and Additional file 1: Fig. S3B), we noticed that two non-*K. pneumoniae* ST307 isolates were also positive for plasmid 1 (NDM-1): (i) PBIO1963 is an *E. coli* ST362 isolate obtained on September 10, 2019, from patient PT07, who also carried *K. pneumoniae* ST307 (PBIO1955—obtained on July 24, 2019). We suggest that *K. pneumoniae* ST307 donated plasmid 1 to PBIO1963 within the patient. (ii) PBIO1961 is a *K. pneumoniae* ST395 isolate. We did not obtain any additional carbapenem-resistant isolates from this patient (PT12), which indicates inter-patient transfer of resistance plasmids (Fig. 3); however, our epidemiologic data do not support this suggestion as no other ST307-positive patient was simultaneously present on the same ward. Plasmids 2 and 4 were also present in some non-ST307 genomes. *K. pneumoniae* ST405 (PBIO1979) isolated on September 25, 2019, from patient PT21 carried plasmid 2 (CTX-M-15). From the same patient, we isolated *K. pneumoniae* ST307 (PBIO1936) on September 18, 2019, again suggesting plasmid intra-bacterial species transfer within this patient. Plasmid 4 (OXA-48) was present in *K. pneumoniae* ST147 (PBIO1999—isolated from patient PT16 on October 2, 2019) and a *K. pneumoniae* ST307 isolate (PBIO2000) obtained from the same patient on September 4, 2019. Plasmid 4 was further carried by PBIO1966, a *C. freundii* ST153 isolate obtained on September 20, 2019, from patient PT02, who additionally carried two *K. pneumoniae* ST307 isolates (PBIO1958: August 5, 2019; PBIO1932: September 14, 2019). It was also present in *E. cloacae* ST45 (PBIO2014: December 24, 2019), which was the only sequenced isolate from patient PT20. Again, these are examples for putative inter-bacterial plasmid transfers within a patient and among patients, respectively (Fig. 3). Inter-patient transfer for PT20 is further supported by the epidemiologic data: PBIO2012 (ST307) was isolated on December 16, 2019, from a putative donor patient (PT10), who stayed on the same ward as PT20.

### Phenotypic experiments

We then investigated the hypothesis that *K. pneumoniae* ST307 outbreak isolates combined high-level antibiotic resistance with fitness, resilience, and virulence features. In several phenotypic experiments, which were selected based on their relevance in the clinical setting and on the presence of resistance and virulence genes in ST307, we investigated five *K. pneumoniae* ST307 isolates from different time points during the outbreak and different patients and hospitals (*outbreak* isolates), two *K. pneumoniae* ST395, and one *K. pneumoniae* ST405 (subsequently termed as the *internal* group). These were compared to four *external K. pneumoniae* isolates with ST498, ST15, ST307, and ST86 including an archetypal, hypervirulent *K. pneumoniae* strain (hvKP1), which are unrelated to the outbreak. In addition, we included a partially plasmid-cured variant (PCV1935) and *controls* for each phenotypic experiment.

**Fig. 4:**
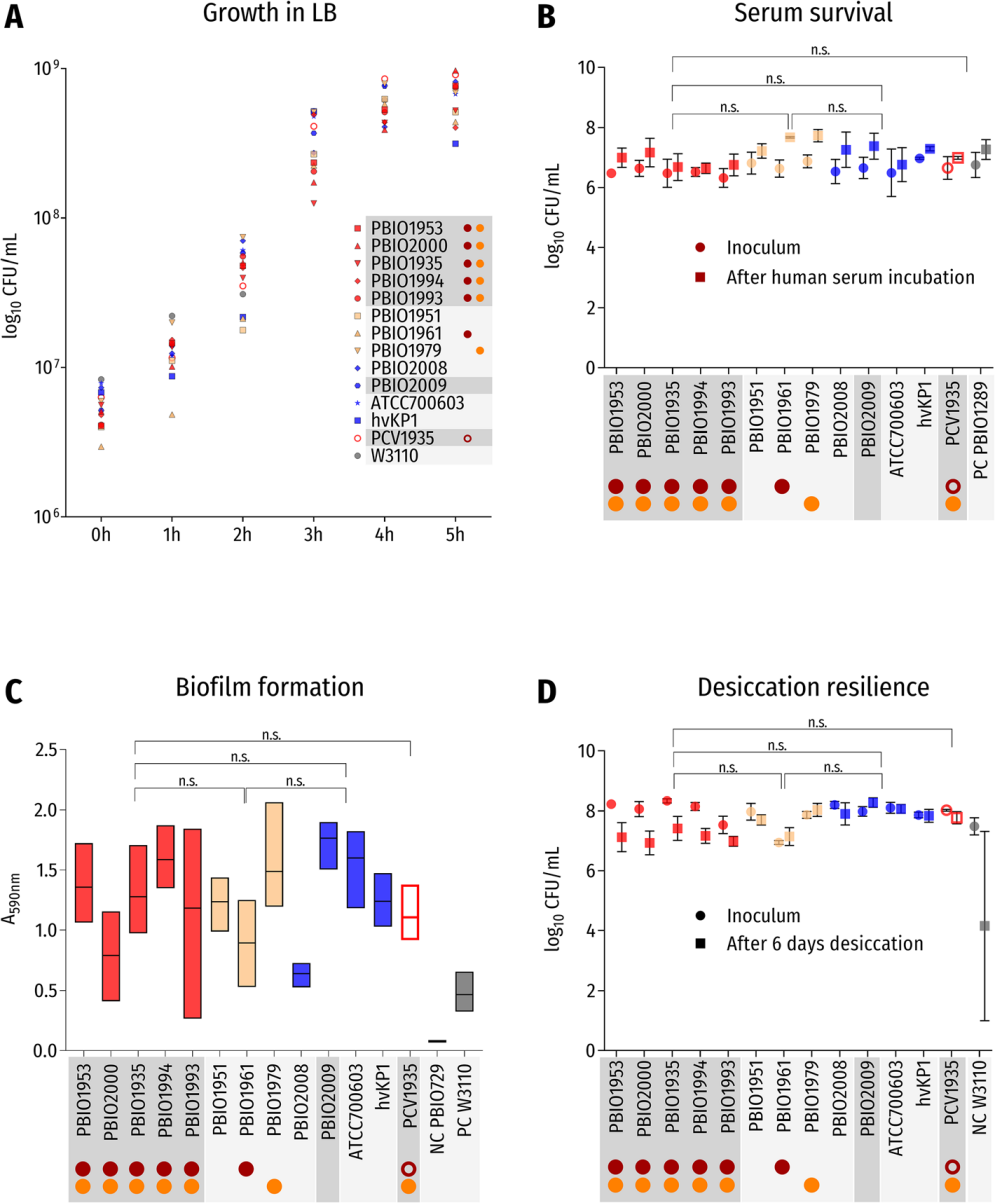
Results of phenotypic experiments without significant differences. Red symbols: *K. pneumoniae* ST307 *outbreak* isolates; yellow symbols: *K. pneumoniae* non-ST307 *internal* isolates; blue symbols: *K. pneumoniae external* isolates. Red circle: occurrence of plasmid 1 in *K. pneumoniae* ST307 *outbreak* isolates and PBIO1961; orange circle: occurrence of plasmid 2 in *K. pneumoniae* ST307 *outbreak* isolates, PBIO1979, and PCV1935; red open circle: incomplete plasmid 1 in PCV1935. Isolates belonging to ST307 are highlighted in dark gray, and all other isolates in light gray. **a** Results of growth experiments in LB medium are given as mean values of CFU/mL for each isolate over 5 h. Abbreviations: n.s., not significant. **b** Results of serum survival experiments are given as mean values and standard deviation of CFU/mL for each isolate before (inoculum: circles) and after 4 h of incubation in human serum (squares). **c** Results of biofilm formation experiments are given as absorbance values (mean with coefficient of variation) of crystal violet at 590 nm for each isolate after 3 h of incubation. **d** Results of desiccation tolerance experiments are given as mean values and standard deviation of colony-forming units (CFU) per milliliter (CFU/mL) for each isolate before (inoculum: circles) and after 6 days of desiccation (squares)

We observed no significant decreased growth rates (*p* > 0.1 at 1, 2, 3, 4, and 5 h) of the ST307 isolates when compared to the internal and external groups and to the *E. coli* K-12 control (Fig. 4a), and also among each other. PCV1935, which carried the incomplete NDM-1 plasmid 1, also showed comparable growth (μ_1h_ST307, 1.05, vs. μ_1h_PCV1935, 0.67; *p* = 0.9999). Interestingly, *K. pneumoniae* ST395 (PBIO1961) demonstrated a prolonged lag phase (μ_1h_ST307, 1.05, vs. μ_1h_PBIO1961, 0.51; *p* = 0.038), which will be further explored in a prospective study.

**Fig. 5:**
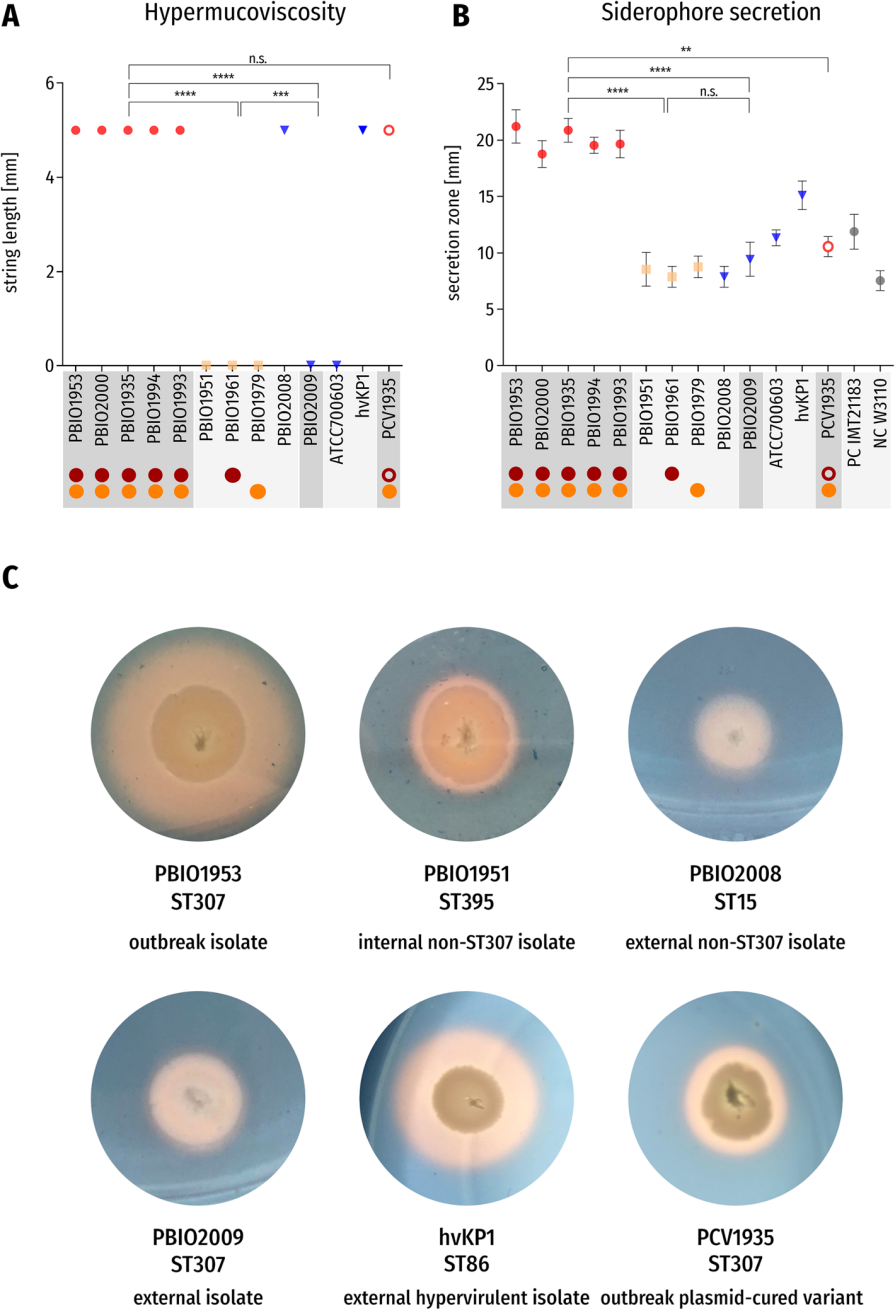
Results of phenotypic experiments with significant differences. Red symbols: *K. pneumoniae* ST307 *outbreak* isolates; yellow symbols: *K. pneumoniae* non-ST307 *internal* isolates; blue symbols: *K. pneumoniae external* isolates. Red circle: occurrence of plasmid 1 in *K. pneumoniae* ST307 *outbreak* isolates and PBIO1961; orange circle: occurrence of plasmid 2 in *K. pneumoniae* ST307 *outbreak* isolates, PBIO1979, and PCV1935; red open circle: incomplete plasmid 1 in PCV1935. Isolates belonging to ST307 are highlighted in dark gray, and all other isolates in light gray. **a** Results of the hypermucoviscosity test are given as mean values and standard deviation of the string length in millimeters (mm) for each isolate. **b** Results of siderophore secretion tests are given as mean values and standard deviation of the secretion zone diameter in millimeters (mm) for each isolate. **c** Siderophore secretion experiment on CAS-Agar of six exemplary isolates: PBIO1953 (*outbreak* isolate [ST307]), PBIO1951 (*internal* control [ST395]), PBIO2008 (*external* control [ST15]), PBIO2009 (*external* control [ST307]), hvKP1 (*external* hypervirulent control [ST86]), and PCV1935 (*outbreak* plasmid-cured variant [ST307]). Yellow areas around colonies indicate siderophore secretion. Abbreviations and symbols: n.s., not significant; ****p value < 0.0001; ***p value < 0.001; **p value < 0.01

Hypermucoviscosity experiments revealed that all *K. pneumoniae* ST307 outbreak isolates showed strong hypermucoviscosity (≥ 5 mm) (Fig. 5a), whereas internal *K. pneumoniae* ST395 and ST405 isolates did not (*p* < 0.0001). Also, the external isolates showed a negative hypermucoid phenotype, with the exception of *K. pneumoniae* ST15 and, unsurprisingly, hvKP1. Hypermucoviscosity of PCV1935 was not significantly different from wildtype PBIO1935, which is interesting given that both plasmid-encoded *rmpA* and *rmpA2* were lost during the curing process*.* On the contrary, PBIO1961, which carried plasmid 1 (Figs. 3, 4, and 5, and Additional file 1: Figure S3B and S7B), did not show hypermucoviscosity. This genome demonstrated a different *rmpA2*-truncation and K locus in comparison to the ST307 clone (Additional file 2: Table S1). Also consider that the different phenotypes might be due to synergy-dependent processes such that plasmids have reduced impacts on other genetic backgrounds than ST307. The hypermucoid phenotype appears to be a fine-tuned process. A recent study [68] showed that loss of *rmpC*, which is a newly identified gene that contributes to capsule regulation in hvKp, resulted in decreased capsule gene expression while simultaneously retaining hypermucoviscosity. Additional investigations will have to address further which regulatory mechanisms contribute to the hypermucoid phenotype in our outbreak clone. Hypermucoviscosity is associated with invasive and other aggressive types of infection, but recent literature suggests that this characteristic alone is not per se responsible for a hypervirulent phenotype in *Klebsiella* spp. and that both terms should not be used synonymously [5, 69].

Similar to the hypermucoviscosity experiments, we observed significant differences between the *K. pneumoniae* ST307 outbreak isolates and the internal and external groups regarding their siderophore secretion capacities (*p* < 0.0001) (Fig. 5b, c). They all exhibited a significant higher secretion with average bleaching zone diameters of 20 mm, compared to 8.4 mm of internal and 10.8 mm of external isolates (positive control, 12 mm). This is likely due to the presence of the NDM-1-plasmid-encoded aerobactin, underlined by the results shown for PCV1935, which demonstrated significantly reduced siderophore secretion (*p* = 0.0025) (Fig. 5b, c). Note that hvKP1 also showed increased siderophore secretion compared to the internal group (*p* = 0.0126) but in tendency less than the ST307 outbreak isolates, although this difference was not significant (*p* = 0.35). PBIO1961 registered within the result range of internal and external isolates. This is likely due to a missense mutation of plasmid-encoded *iutA* and/or the different character of yersiniabactin (“unknown” ybt—Additional file 2: Table S1). Given that hypermucoviscosity, aerobactin secretion, and the metabolite transporter PEG344 [70] are suggested key virulence features of hypervirulent *Klebsiella* strains during infection, the above-average performance, in addition to extensive antibiotic resistance expression, provides one step closer to explaining why this outbreak evolved. PBIO2009 (external ST307 isolate), which does not possess typical hvKp-associated features like *peg-344*, *iucA*, and *rmpA* [11], showed negative hypermucoid and iron uptake phenotypes (Fig. 5a–c), possibly strengthening our assumption.

We were then interested in whether the *K. pneumoniae* ST307 outbreak isolates also showed sufficient capacity for survival and resilience in the clinical setting and host. Serum survival experiments revealed high survival rates for all isolates, and together with similar strong biofilm formation capacities (Fig. 4b, c), this suggests that the outbreak *K. pneumoniae* ST307 isolates had good abilities to resist clinical challenges. Comparable results were also obtained for desiccation resilience (Fig. 4d). Despite that internal and external groups showed a tendency to survive 6 days of desiccation at higher rates than *K. pneumoniae* ST307, the difference was not significant after applying Bonferroni correction (outbreak vs. internal group: *p* = 0.5488; outbreak vs. external group: *p* = 0.0730; internal vs. external group: *p* > 0.9999).

The genetic characterization revealed several heavy metal efflux genes on plasmid 2. Heavy metal compounds, such as zinc oxide or copper sulfate, are regularly used as feed supplements in livestock, e.g., for prevention of gastro-intestinal disorders and growth promotion in piglets [71], and co-selection of heavy metal and antimicrobial resistance has been increasingly reported [72, 73]. We thus investigated the bacteria’s tolerance by determining minimum inhibitory concentrations (MICs) to copper, zinc, and silver (Additional file 2: Table S1). A MIC value of 1024 μg/mL for copper sulfate was obtained for almost all *K. pneumoniae* isolates, both outbreak, internal, and external group isolates (control ATCC25922, 256 μg/mL), which is an average copper tolerance for *Enterobacteriaceae* [74]. Interestingly, hvKP1 showed a reduced MIC for copper sulfate (256 μg/mL). We observed similar results for zinc and silver for all isolates (MIC zinc chloride 512 μg/mL, control 256 μg/mL; silver nitrate 4 μg/mL, control 4 μg/mL), again with the exception of hvKP1 demonstrating a reduced MIC for zinc (256 μg/mL).

## Discussion

To the best of our knowledge, this is the first outbreak in Germany of a *K. pneumoniae* ST307 clone that produced both NDM-1 and OXA-48 carbapenemases and showed resistance against colistin. It was first detected at the University Medicine Greifswald in June 2019 from a tracheal secretion sample [19]. As we did not detect any *mcr*-genes, we suggest that colistin resistance is due to chromosomal point mutations including the two-component systems PhoP/PhoQ and PmrA/PmrB, which have been previously described in this context [67].

When placing our outbreak clone in a global frame, a cluster of KPC-producing ST307 genomes from the UK was the phylogenetically closest. Interestingly, this cluster was part of a study from 2017 revealing that these genomes harbor genetic features important for clinical and host adaptation, in particular glycogen synthesis [3]. Our outbreak isolates have seemingly developed different resistance phenotypes and virulence strategies, and the UK cluster is thus probably not the true, most likely recent common ancestor of the ST307 outbreak clone.

Although we did not unequivocally verify the hypervirulent character of this clone, it demonstrated hypermucoviscosity, iron uptake, and metabolite transporter capacities—which are relevant for invasive infection and assertiveness in different host environments [41, 69, 70]—comparable to an archetypal hvKp strain [54]. The fact that these key hypervirulence features in addition to disinfectant resistance are found on mutual virulence/resistance plasmids in extensively drug-resistant isolates is concerning and has tremendous public health implications as these mobile genetic elements may be transferred across different bacteria [75]. Our previous work, and those of others, suggests that the combination of high-level drug resistance and virulence is a good combination for the successful spread of bacterial pathogens [3, 53, 76–79]. On the other hand, the co-carriage of plasmid-encoded heavy metal efflux genes did not significantly impact the phenotypic tolerance in the study’s *K. pneumoniae* outbreak isolates, pointing towards that this capacity is less likely a major contributor to the clone’s success in this outbreak situation.

We detected identical plasmids among ST307 and other *K. pneumoniae* isolates as well as other bacterial genera, exacerbating the threat this clone poses across clinical settings. Note, however, that it is possible that ST307 has a greater tolerance towards possible fitness costs of the carried plasmids, implying that donated plasmids might have reduced impacts in other genetic backgrounds [56]. We suggest that the *K. pneumoniae* ST307 isolates are the general plasmid donors; they were all isolated at an earlier date than the putative acceptor isolates and more independent variants accumulated in some putative acceptor plasmids compared to the *K. pneumoniae* ST307 donor plasmids (Additional file 2: Table S3). The fact that ST395 occurred three times among all isolates but only ST395 PBIO1961 was positive for plasmid 1 additionally strengthens this assumption.

Interestingly, although most hvKp show susceptibility to antimicrobials [44], hvKp with AMR have increasingly emerged in the last decade [80–83], which might be an ongoing trend.

## Conclusions

While the emergence of XDR, virulent, and fit pathogens is worrisome, our study helps to implement control measures and calls for prospective surveillance strategies that take the emergence of “converging” cKp and hvKp pathotypes into account.

## Supplementary Information

**Additional file 1: Fig. S1.** Long-read alignment for plasmid 1 (pPBIO1953_NDM-1). **Fig. S2.** Assembly graphs of the reference isolate PBIO1953 and the putative plasmid recipient isolates. **Fig. S3.** Plasmidogram of different bacterial isolates. **Fig. S4.** Global core SNP phylogeny of *K. pneumoniae* ST307 isolates. **Fig. S5.** Single-nucleotide polymorphism (SNP) distribution of *K. pneumoniae* ST307 isolates. **Fig. S6.** Missense variants in genes putatively related to colistin resistance. **Fig. S7.** Comparison of the closed genome of PBIO1953 (PT18) with the other isolates.

**Additional file 2: Table S1.** Overview of strains investigated in this study. **Table S2.** Overview of publicly available *Klebsiella pneumoniae* ST307 genomes obtained from online sources. **Table S3.** Overview of accumulated variants in transferred plasmids. **Table S4.** Coverage of isolate replicons.

## Data Availability

The experimental and computational data that support the findings of this research are available in this article and its supplementary information files. The genomic data have been deposited in the European Nucleotide Archive (ENA) at EMBL-EBI under accession number PRJEB37933 (https://www.ebi.ac.uk/ena/browser/view/PRJEB37933) [84].

## Abbreviations

MDR: Multidrug-resistant
XDR: Extensively drug-resistant
cKp: Classical *K. pneumoniae*
hvKp: Hypervirulent *K. pneumoniae*
ST: Sequence type
MLST: Multi-locus sequence typing
PCV: Plasmid-cured variant
CFU: Colony-forming units
SNP: Single-nucleotide polymorphism
CDS: Coding sequence
UK: United Kingdom
