# Supplementary material for: A Klebsiella pneumoniae ST307 outbreak clone from Germany demonstrates features of extensive drug resistance, hypermucoviscosity, and enhanced iron acquisition
Source: Genome Med. 2020 Dec 9;12:113. doi: 10.1186/s13073-020-00814-6 (PMC7724794; doi:10.1186/s13073-020-00814-6)
Supplement: Supplementary file 1 — Additional file 1: Fig. S1. Long-read alignment for plasmid 1 (pPBIO1953_NDM-1). Fig. S2. Assembly graphs of the reference isolate PBIO1953 and the putative plasmid recipient isolates. Fig. S3. Plasmidogram of different bacterial isolates. Fig. S4. Global core SNP phylogeny of K. pneumoniae ST307 isolates. Fig. S5. Single-nucleotide polymorphism (SNP) distribution of K. pneumoniae ST307 isolates. Fig. S6. Missense variants in genes putatively related to colistin resistance. Fig. S7. Comparison of the closed genome of PBIO1953 (PT18) with the other isolates. [file 13073_2020_814_MOESM1_ESM.pdf]

Additional file 1

A *Klebsiella pneumoniae* ST307 outbreak clone from Germany demonstrates features of extensive drug resistance, hypermucoviscosity and enhanced iron acquisition

Stefan E. Heiden[+], Nils-Olaf Hübner[+], Jürgen A. Bohnert, Claus-Dieter Heidecke, Axel Kramer, Veronika Balau, Wolfgang Gierer, Stephan Schaefer, Tim Eckmanns, Sören Gatermann, Elias Eger, Sebastian Guenther, Karsten Becker, Katharina Schaufler[*]

[+]shared first authorship, [*]corresponding author

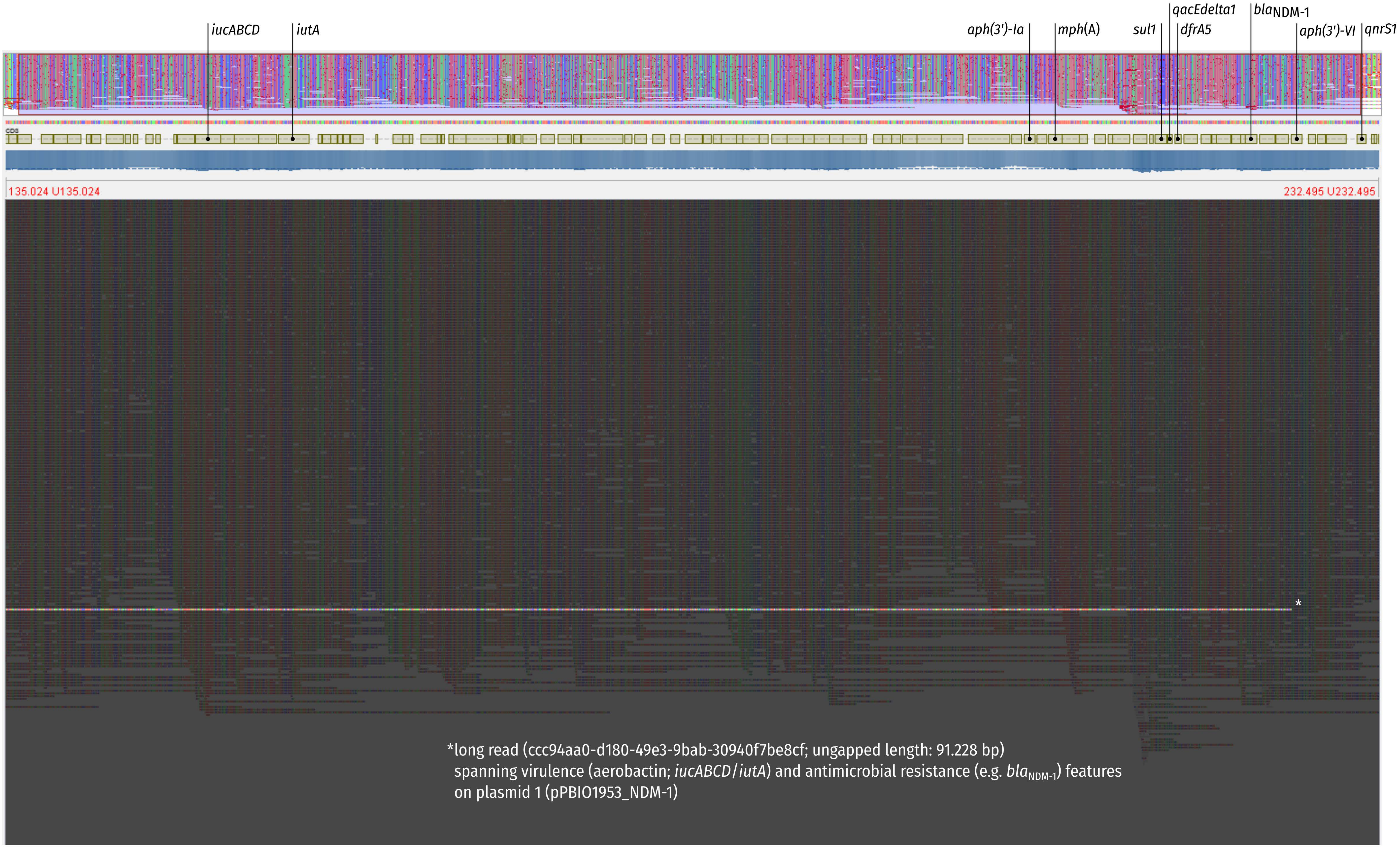
iucABCD
iutA
aph(3')-Ia
mph(A)
sul1
qacEdelta1
dfrA5
blaNDM-1
aph(3')-VI
qnrS1
CDS
135.024 U135.024
232.495 U232.495
*
*long read (ccc94aa0-d180-49e3-9bab-30940f7be8cf; ungapped length: 91.228 bp)
spanning virulence (aerobactin; iucABCD/iutA) and antimicrobial resistance (e.g. blaNDM-1) features
on plasmid 1 (pPBIO1953_NDM-1)

Fig. S1. Long-read alignment for plasmid 1 (pPBIO1953_NDM-1). The highlighted long read has a gapless length of 91.228 bp and spans both virulence (aerobactin; *iucABCD*/*iutA*) and antimicrobial resistance (e.g. bla_{NDM-1}) features. The homogeneous distribution of the reads is apparent.

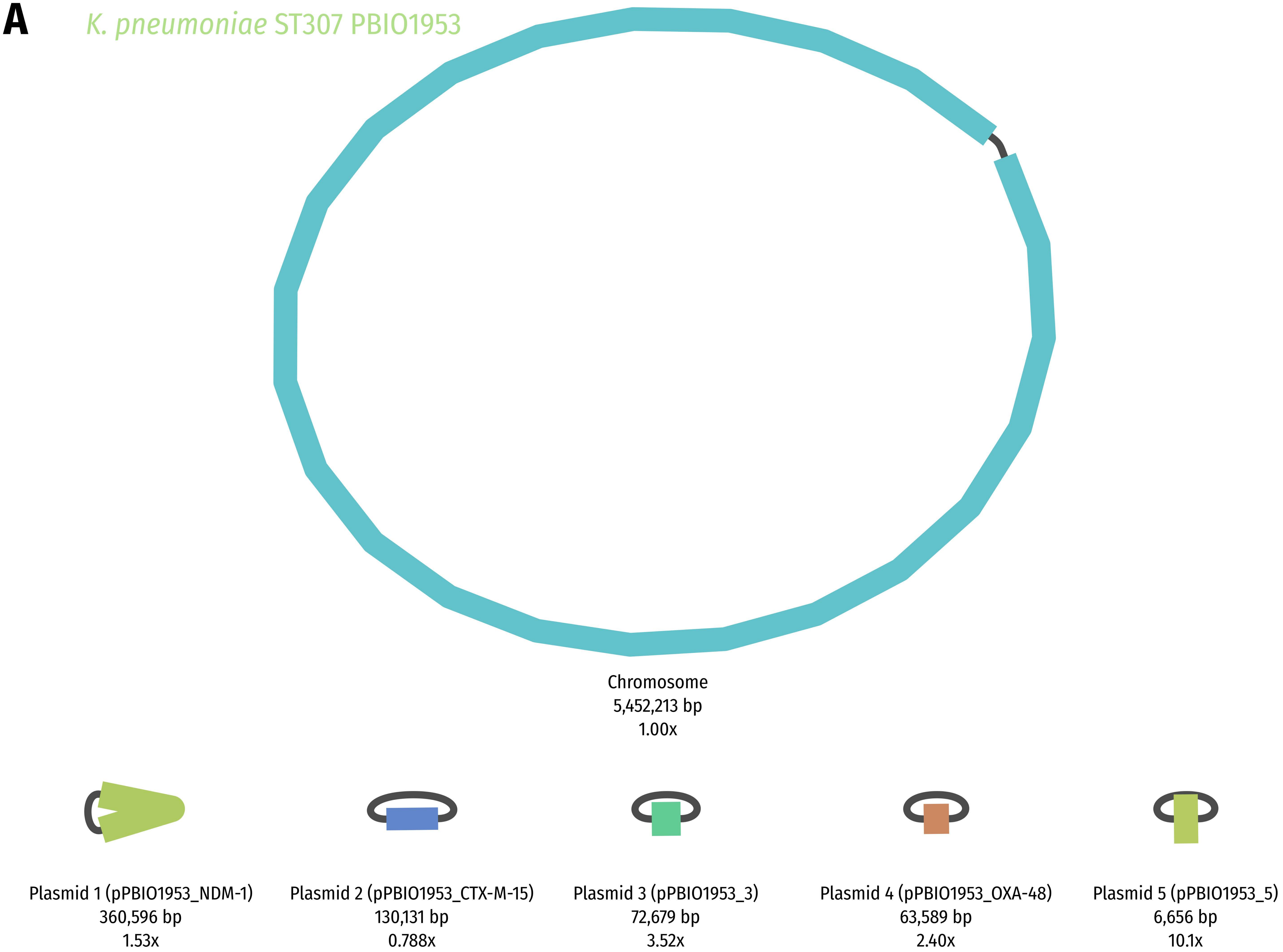
A
K. pneumoniae ST307 PBIO1953
Chromosome
5,452,213 bp
1.00x
Plasmid 1 (pPBIO1953_NDM-1)
360,596 bp
1.53x
Plasmid 2 (pPBIO1953_CTX-M-15)
130,131 bp
0.788x
Plasmid 3 (pPBIO1953_3)
72,679 bp
3.52x
Plasmid 4 (pPBIO1953_OXA-48)
63,589 bp
2.40x
Plasmid 5 (pPBIO1953_5)
6,656 bp
10.1x

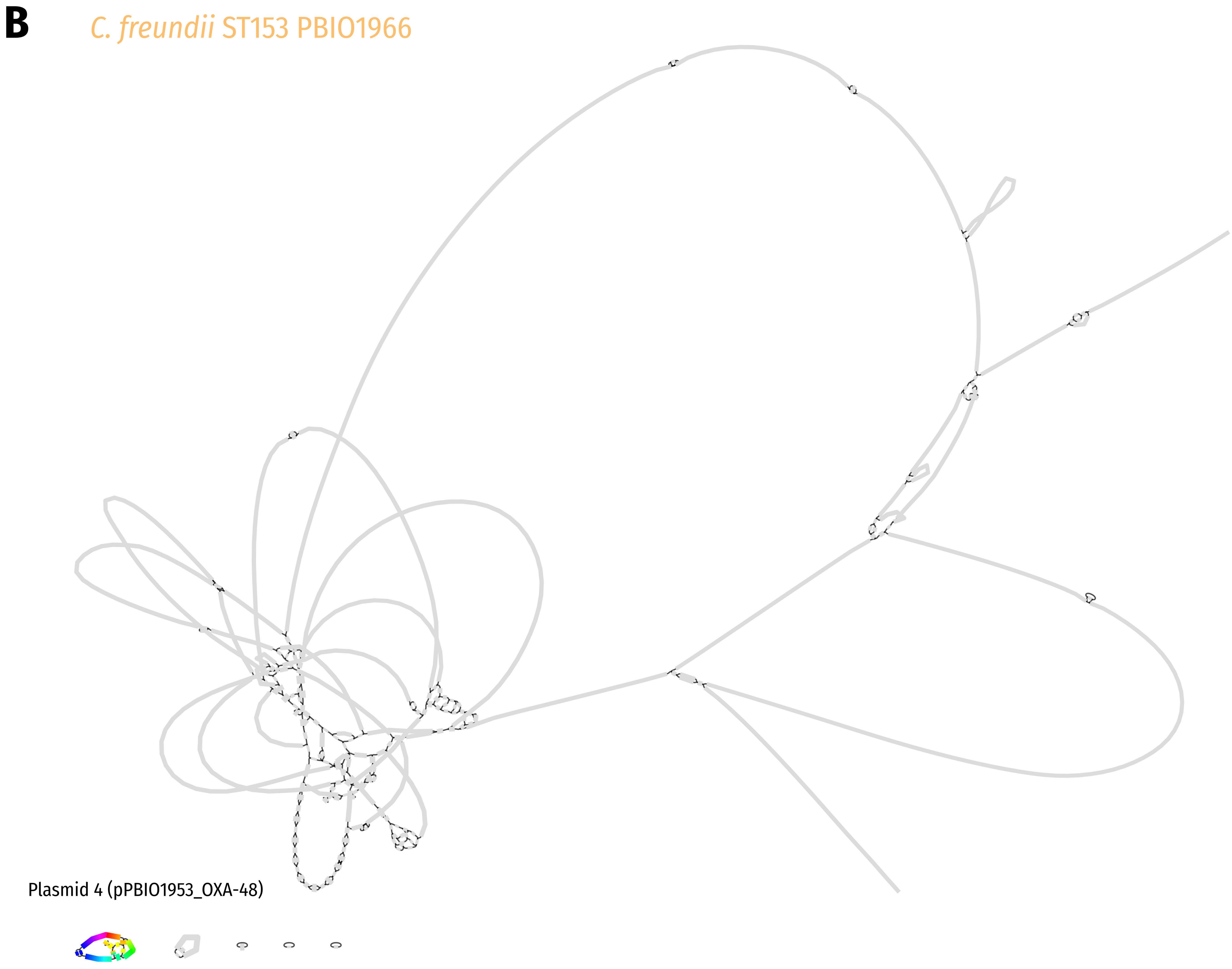
B
C. freundii ST153 PBIO1966
Plasmid 4 (pPBIO1953_OXA-48)

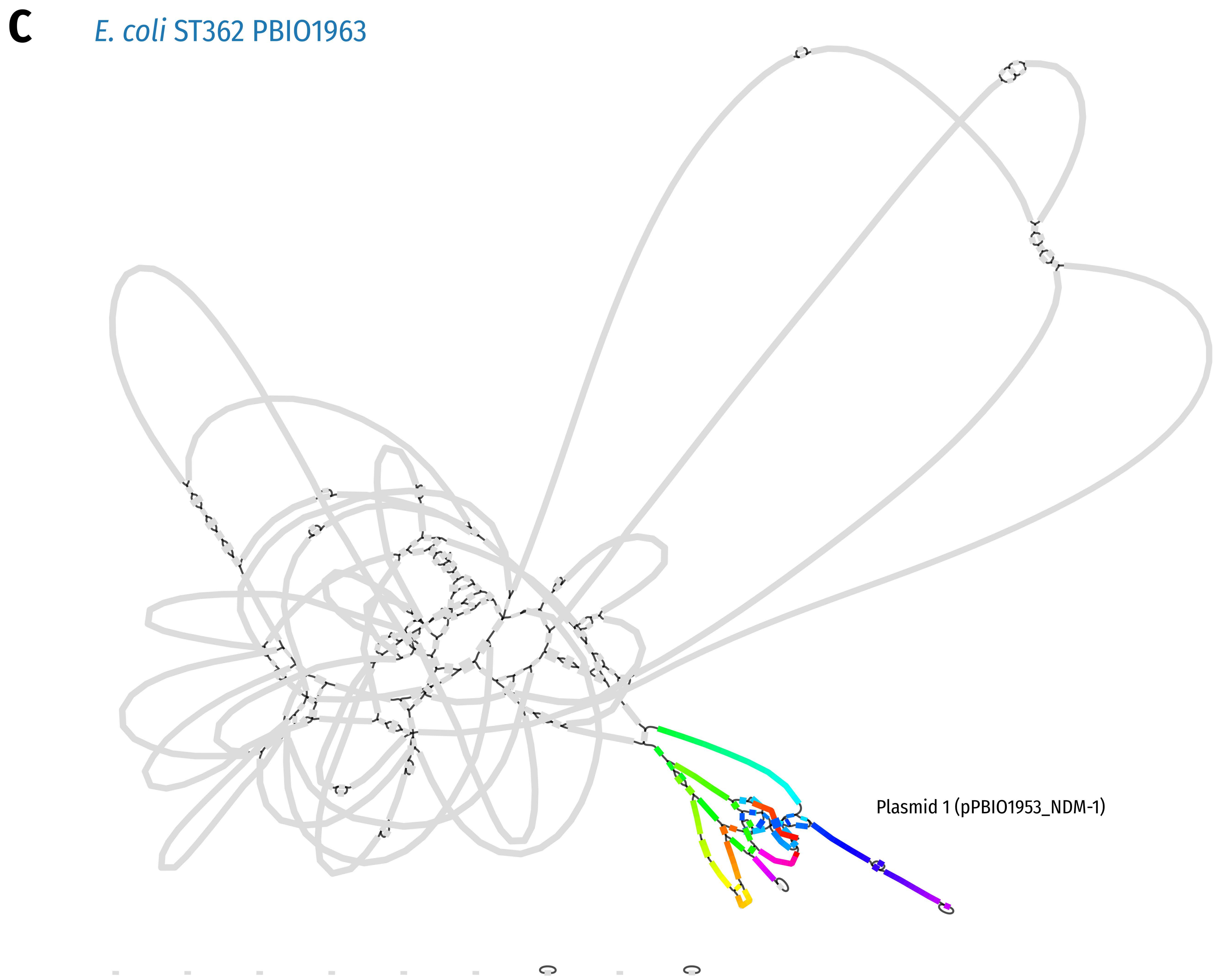
C
E. coli ST362 PBIO1963
Plasmid 1 (pPBIO1953_NDM-1)

D *E. cloacae* ST45 PBIO2014

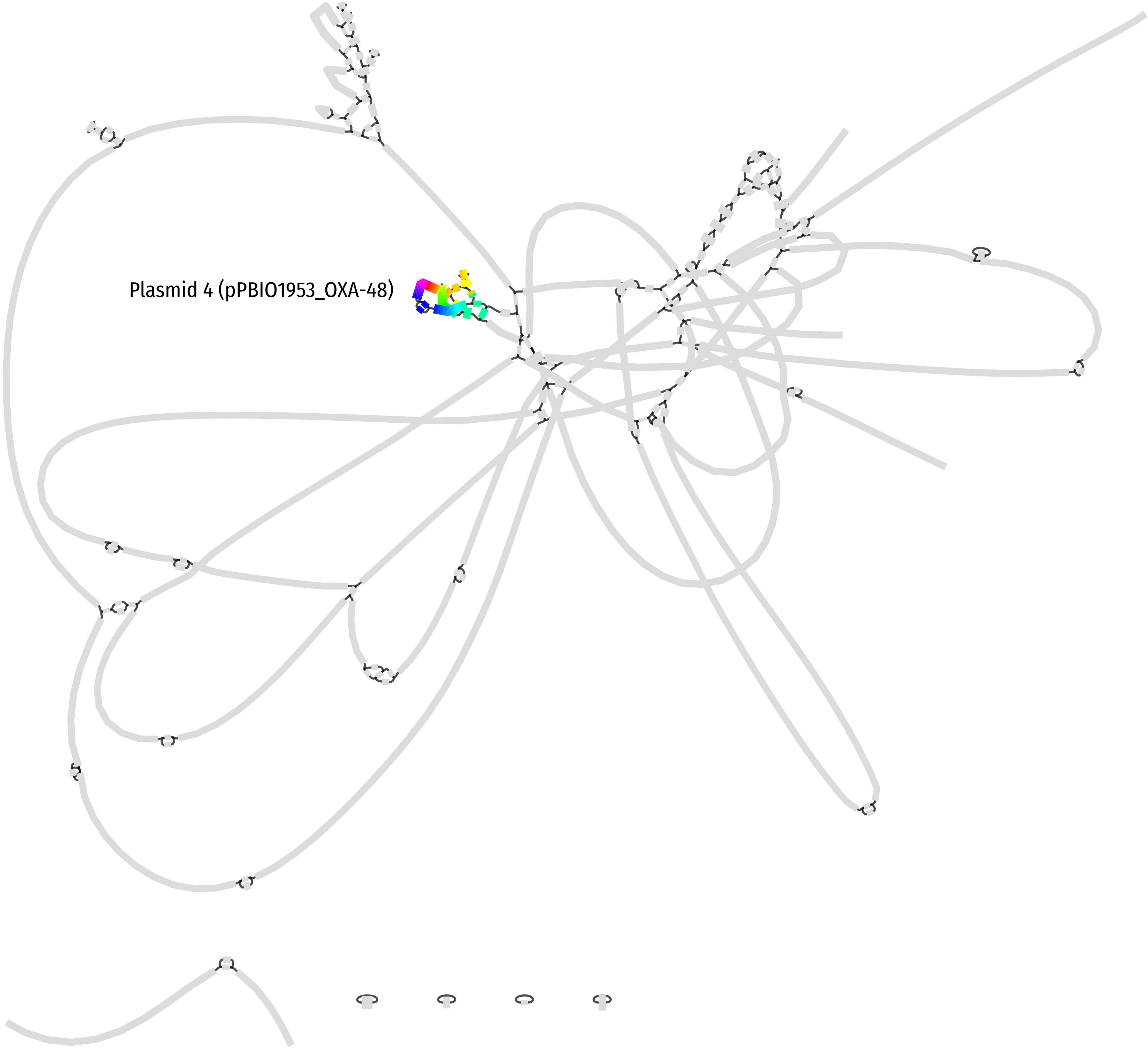

E

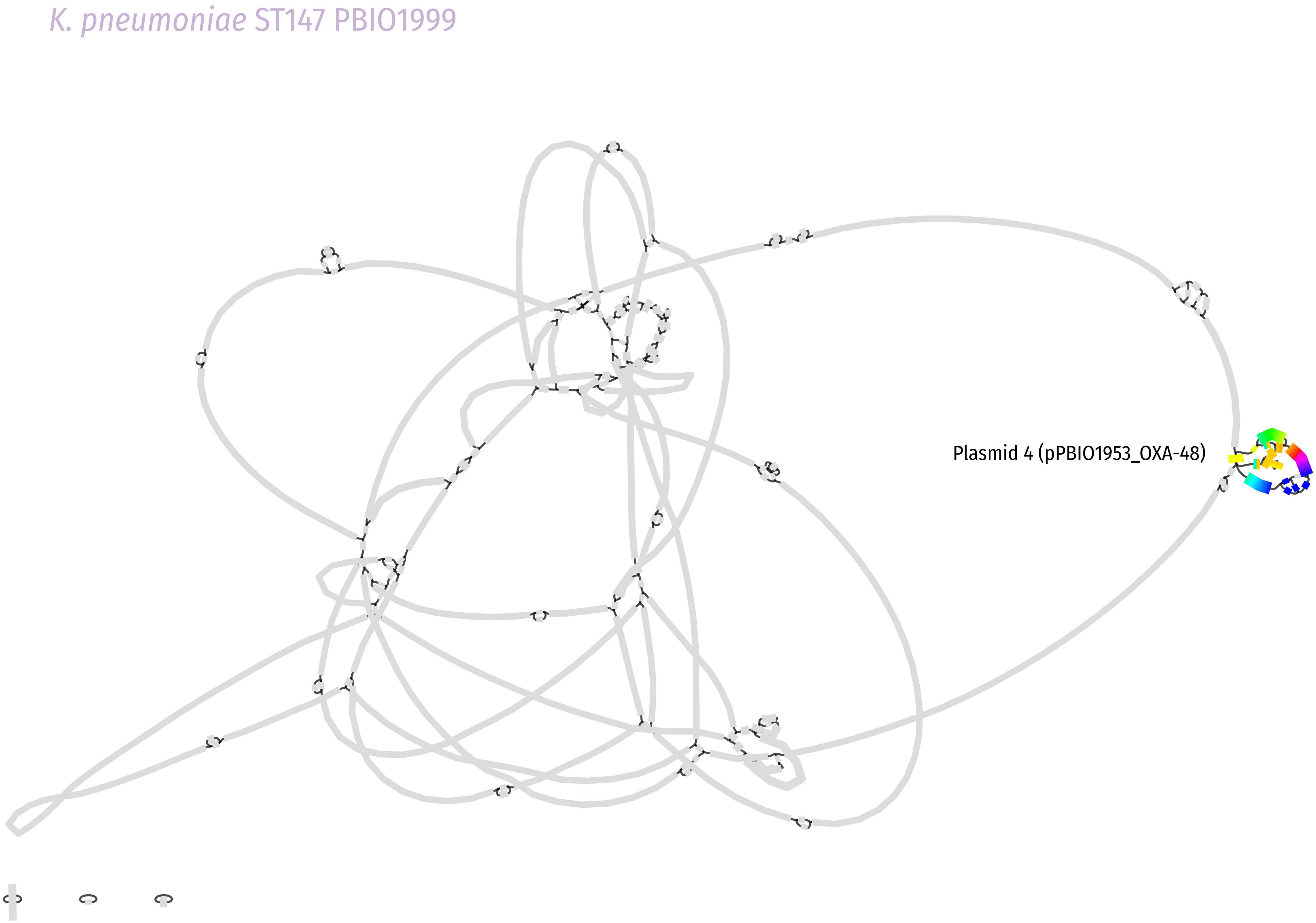

F

K. pneumoniae ST405 PBIO1979

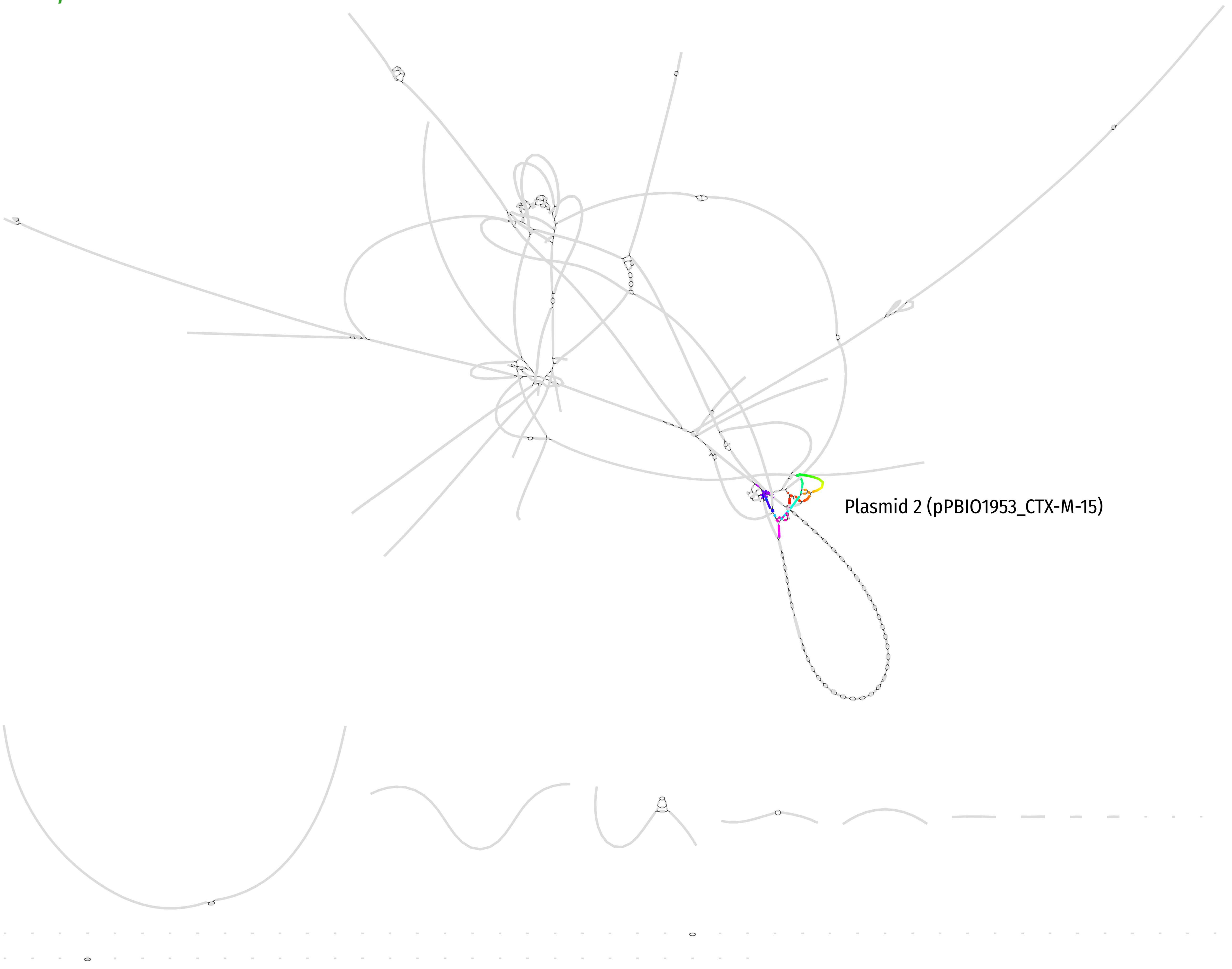

G

K. pneumoniae ST395 PBIO1961

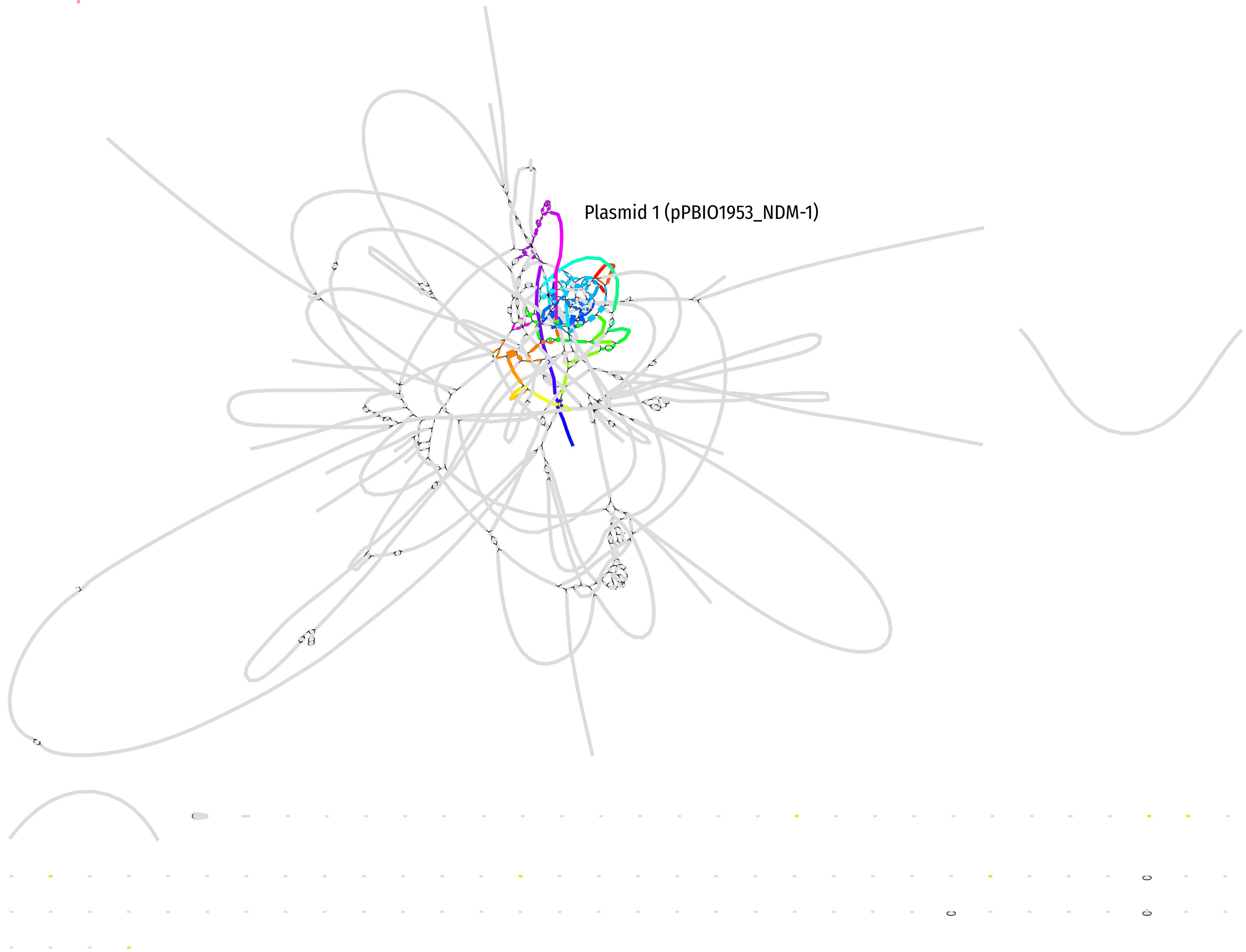

H *K. pneumoniae* ST307 PBIO1953

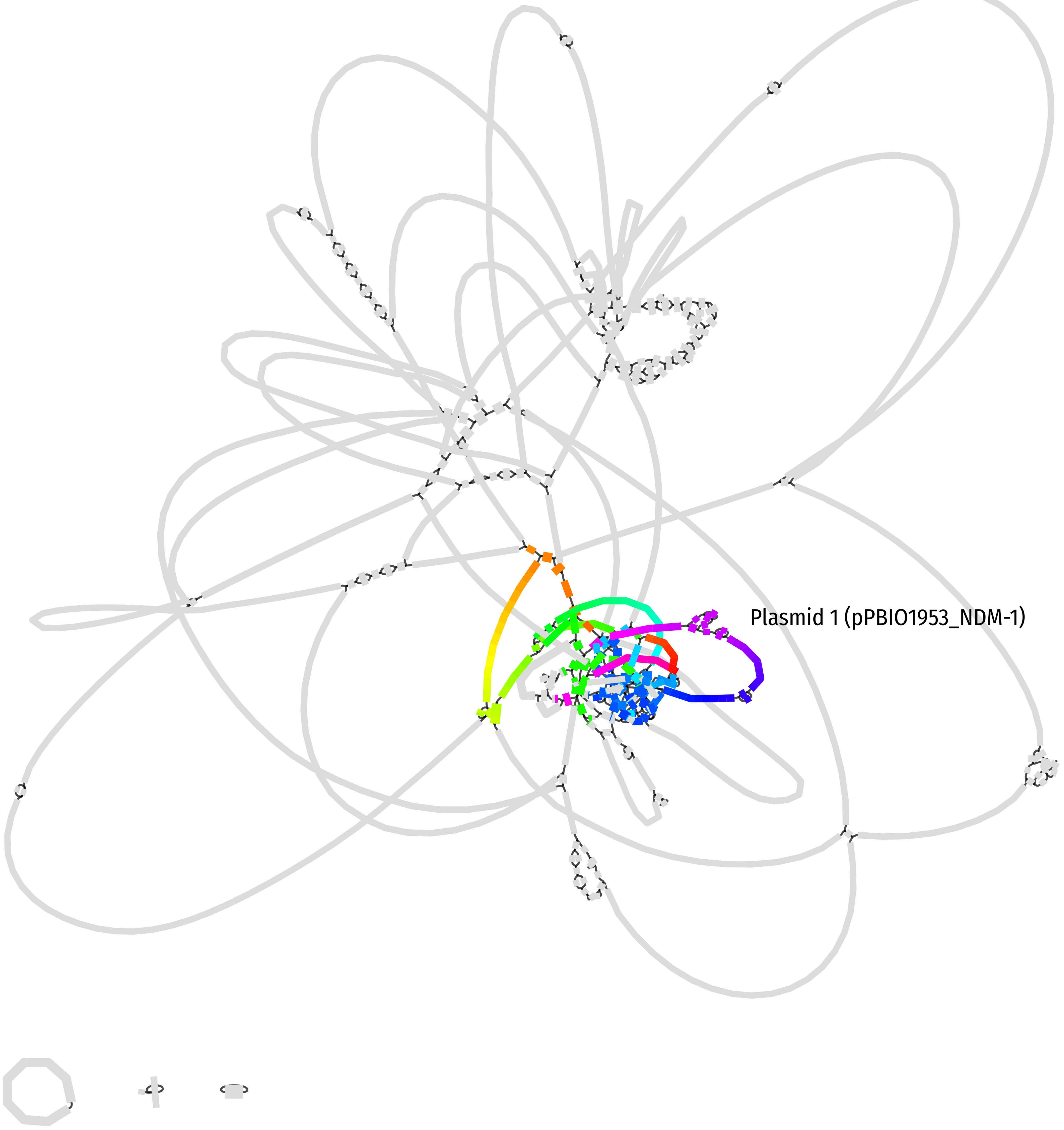

Fig. S2. Assembly graphs of the reference isolate PBIO1953 (A: hybrid assembly; H: short-read assembly) **and the putative plasmid recipient isolates** PBIO1966 (B: *C. freundii* ST153, plasmid 4), PBIO1963 (C: *E. coli* ST362, plasmid 1), PBIO2014 (D: *E. cloacae* ST45, plasmid 4), PBIO1999 (E: Kp ST147, plasmid 4), PBIO1979 (F: Kp ST405, plasmid 2), and PBIO1961 (G: Kp ST395, plasmid 1). In B-H, BLAST results (Megablast, >= 99 % identity, E-value 1e-10) for the labeled plasmids are shown with a rainbow coloring. Note that in all graphs but F and G, plasmid contigs are only connected with the rest of the graph by a maximum of one node. The plasmids in F and G are tangled with other contigs as it is the case for the short-read assembly of PBIO1953 (H).

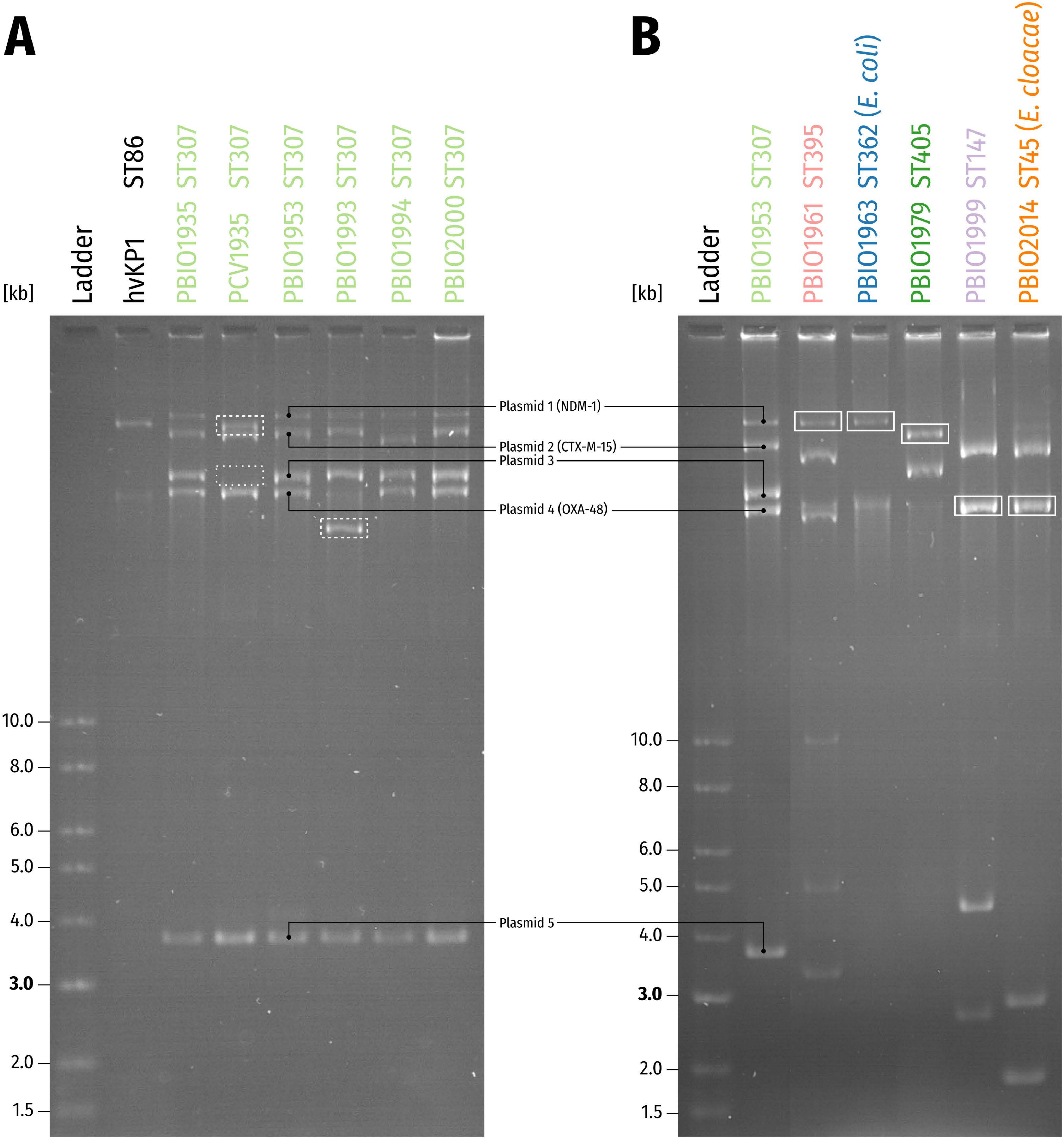

A
B
[kb]
Ladder
hvKP1 ST86
PBIO1935 ST307
PCV1935 ST307
PBIO1953 ST307
PBIO1993 ST307
PBIO1994 ST307
PBIO2000 ST307
PBIO1961 ST395
PBIO1963 ST362 (E. coli)
PBIO1979 ST405
PBIO1999 ST147
PBIO2014 ST45 (E. cloacae)
Plasmid 1 (NDM-1)
Plasmid 2 (CTX-M-15)
Plasmid 3
Plasmid 4 (OXA-48)
Plasmid 5
10.0
8.0
6.0
5.0
4.0
3.0
2.0
1.5

Fig. S3. Plasmidogram of different bacterial isolates. Rectangles indicate the relevant plasmids. A: *K. pneumoniae outbreak* isolates, partially plasmid-cured variant and *external* archetypal hvKP1 strain. From left to right: DNA ladder (largest band: 10 kbp), hvKP1 (Kp ST86), PBIO1935 (Kp ST307), PCV1935 (Kp ST307; size-reduced plasmid 1 [dashed outline] and missing plasmid 3 [dotted outline]), PBIO1953 (Kp ST307), PBIO1993 (Kp ST307; size-reduced plasmid 4 [dashed outline]), PBIO1994 (Kp ST307) and PBIO2000 (Kp ST307). B: *K. pneumoniae* and other bacterial isolates relevant in the plasmid transmission context. From left to right: DNA ladder (largest band: 10 kbp), PBIO1953 (Kp ST307), PBIO1961 (Kp ST395; plasmid 1 [solid outline]), PBIO1963 (*E. coli* ST362; plasmid 1 [solid outline]), PBIO1979 (Kp ST405; plasmid 2 [solid outline]), PBIO1999 (Kp ST147; plasmid 4 [solid outline]) and PBIO2014 (*E. cloacae* ST45; plasmid 4 [solid outline]).

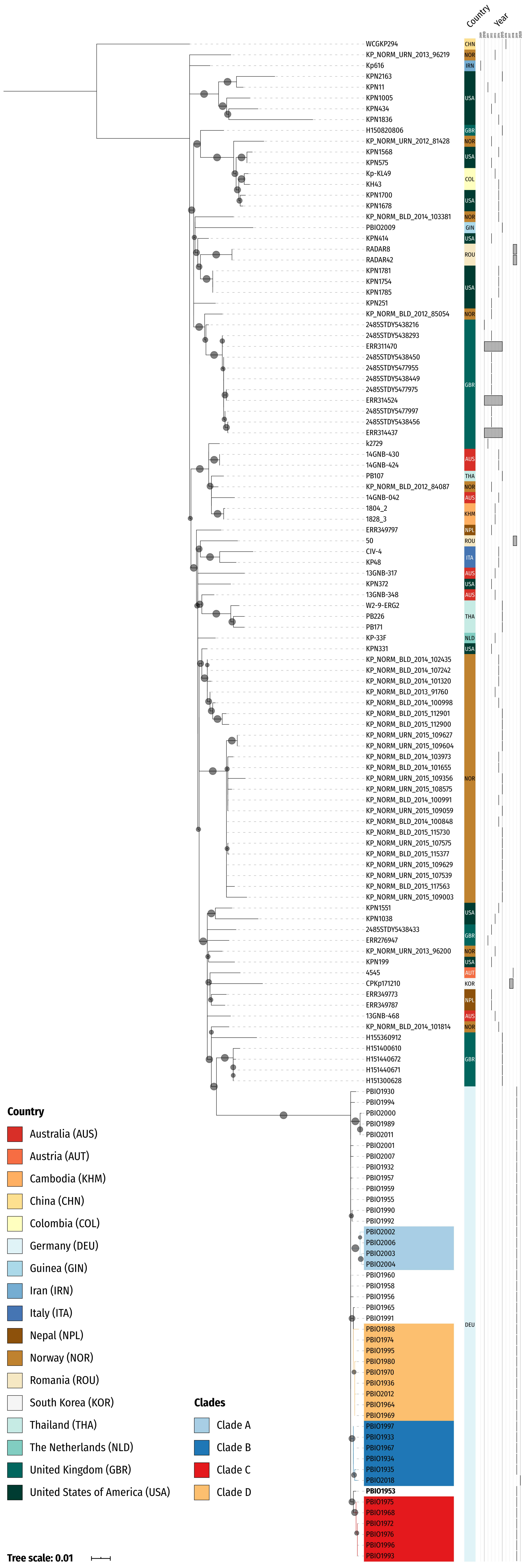

Country
Year
WCGKP294
KP_NORM_URN_2013_96219
Kp616
KPN2163
KPN11
KPN1005
KPN434
KPN1836
H150820806
KP_NORM_URN_2012_81428
KPN1568
KPN575
Kp-KL49
KH43
KPN1700
KPN1678
KP_NORM_BLD_2014_103381
PBIO2009
KPN414
RADAR8
RADAR42
KPN1781
KPN1754
KPN1785
KPN251
KP_NORM_BLD_2012_85054
2485STDY5438216
2485STDY5438293
ERR311470
2485STDY5438450
2485STDY5477955
2485STDY5438449
2485STDY5477975
ERR314524
2485STDY5477997
2485STDY5438456
ERR314437
k2729
14GNB-430
14GNB-424
PB107
KP_NORM_BLD_2012_84087
14GNB-042
1804_2
1828_3
ERR349797
50
CIV-4
KP48
13GNB-317
KPN372
13GNB-348
W2-9-ERG2
PB226
PB171
KP-33F
KPN331
KP_NORM_BLD_2014_102435
KP_NORM_BLD_2014_107242
KP_NORM_BLD_2014_101320
KP_NORM_BLD_2013_91760
KP_NORM_BLD_2014_100998
KP_NORM_BLD_2015_112901
KP_NORM_BLD_2015_112900
KP_NORM_URN_2015_109627
KP_NORM_URN_2015_109604
KP_NORM_BLD_2014_103973
KP_NORM_BLD_2014_101655
KP_NORM_URN_2015_109356
KP_NORM_URN_2015_108575
KP_NORM_BLD_2014_100991
KP_NORM_URN_2015_109059
KP_NORM_BLD_2014_100848
KP_NORM_BLD_2015_115730
KP_NORM_URN_2015_107575
KP_NORM_BLD_2015_115377
KP_NORM_URN_2015_109629
KP_NORM_URN_2015_107539
KP_NORM_BLD_2015_117563
KP_NORM_URN_2015_109003
KPN1551
KPN1038
2485STDY5438433
ERR276947
KP_NORM_URN_2013_96200
KPN199
4545
CPKp171210
ERR349773
ERR349787
13GNB-468
KP_NORM_BLD_2014_101814
H155360912
H151400610
H151440672
H151440671
H151300628
PBIO1930
PBIO1994
PBIO2000
PBIO1989
PBIO2011
PBIO2001
PBIO2007
PBIO1932
PBIO1957
PBIO1959
PBIO1955
PBIO1990
PBIO1992
PBIO2002
PBIO2006
PBIO2003
PBIO2004
PBIO1960
PBIO1958
PBIO1956
PBIO1965
PBIO1991
PBIO1988
PBIO1974
PBIO1995
PBIO1980
PBIO1970
PBIO1936
PBIO2012
PBIO1964
PBIO1969
PBIO1997
PBIO1933
PBIO1967
PBIO1934
PBIO1935
PBIO2018
PBIO1953
PBIO1975
PBIO1968
PBIO1972
PBIO1976
PBIO1996
PBIO1993
CHN
NOR
IRN
USA
GBR
NOR
USA
COL
USA
NOR
GIN
USA
ROU
USA
NOR
GBR
AUS
THA
NOR
AUS
KHM
NPL
ROU
ITA
AUS
USA
AUS
THA
NLD
USA
NOR
USA
GBR
NOR
USA
AUT
KOR
NPL
AUS
NOR
GBR
DEU
Country
Australia (AUS)
Austria (AUT)
Cambodia (KHM)
China (CHN)
Colombia (COL)
Germany (DEU)
Guinea (GIN)
Iran (IRN)
Italy (ITA)
Nepal (NPL)
Norway (NOR)
Romania (ROU)
South Korea (KOR)
Thailand (THA)
The Netherlands (NLD)
United Kingdom (GBR)
United States of America (USA)
Clades
Clade A
Clade B
Clade C
Clade D
Tree scale: 0.01

Fig. S4. Global core SNP phylogeny of *K. pneumoniae* ST307 isolates. Besides the 44 ST307 isolates from this study, the phylogenetic tree depicts 97 publicly available ST307 genomes. The nodes are either labeled by strain, isolate or accession number (Additional file 2: Table S1 and Table S2). The reference isolate (PBIO1953) is shown in bold. The phylogenetic tree was inferred with a Maximum Likelihood-based approach and is based on a core SNP alignment (1476 sites). The (midpoint-rooted) tree is shown with bootstrap proportions (filled circles on branches) for values ≥ 50 % (1000 replicates) and additional metadata.

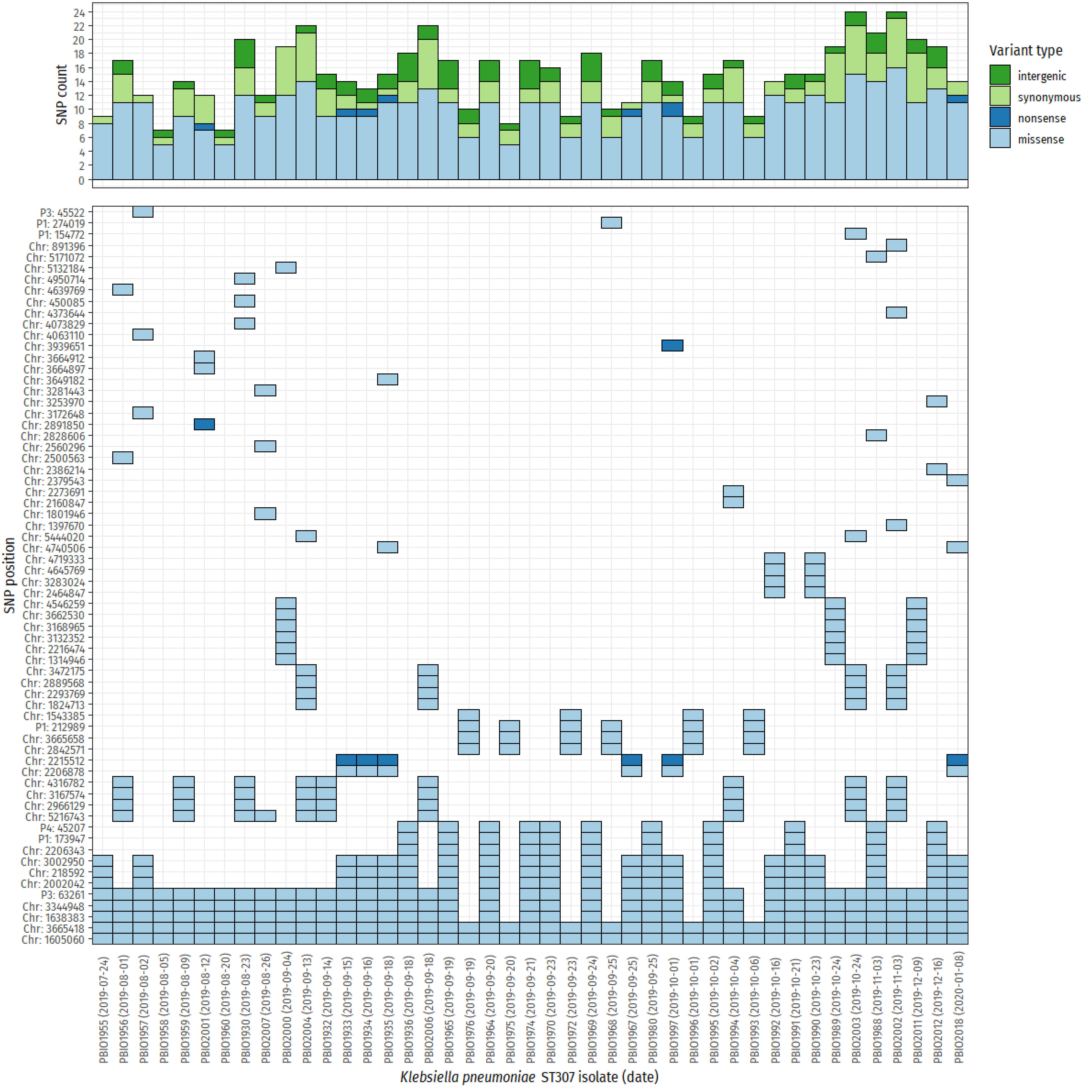
SNP count
Variant type
intergenic
synonymous
nonsense
missense
SNP position
P3: 45522
P1: 274019
P1: 154772
Chr: 891396
Chr: 5171072
Chr: 5132184
Chr: 4950714
Chr: 4639769
Chr: 450085
Chr: 4373644
Chr: 4073829
Chr: 4063110
Chr: 3939651
Chr: 3664912
Chr: 3664897
Chr: 3649182
Chr: 3281443
Chr: 3253970
Chr: 3172648
Chr: 2891850
Chr: 2828606
Chr: 2560296
Chr: 2500563
Chr: 2386214
Chr: 2379543
Chr: 2273691
Chr: 2160847
Chr: 1801946
Chr: 1397670
Chr: 5444020
Chr: 4740506
Chr: 4719333
Chr: 4645769
Chr: 3283024
Chr: 2464847
Chr: 4546259
Chr: 3662530
Chr: 3168965
Chr: 3132352
Chr: 2216474
Chr: 1314946
Chr: 3472175
Chr: 2889568
Chr: 2293769
Chr: 1824713
Chr: 1543385
P1: 212989
Chr: 3665658
Chr: 2842571
Chr: 2215512
Chr: 2206878
Chr: 4316782
Chr: 3167574
Chr: 2966129
Chr: 5216743
P4: 45207
P1: 173947
Chr: 2206343
Chr: 3002950
Chr: 218592
Chr: 2002042
P3: 63261
Chr: 3344948
Chr: 1638383
Chr: 3665418
Chr: 1605060
PBIO1955 (2019-07-24)
PBIO1956 (2019-08-01)
PBIO1957 (2019-08-02)
PBIO1958 (2019-08-05)
PBIO1959 (2019-08-09)
PBIO2001 (2019-08-12)
PBIO1960 (2019-08-20)
PBIO1930 (2019-08-23)
PBIO2007 (2019-08-26)
PBIO2000 (2019-09-04)
PBIO2004 (2019-09-13)
PBIO1932 (2019-09-14)
PBIO1933 (2019-09-15)
PBIO1934 (2019-09-16)
PBIO1935 (2019-09-18)
PBIO1936 (2019-09-18)
PBIO2006 (2019-09-18)
PBIO1965 (2019-09-19)
PBIO1976 (2019-09-19)
PBIO1964 (2019-09-20)
PBIO1975 (2019-09-20)
PBIO1974 (2019-09-21)
PBIO1970 (2019-09-23)
PBIO1972 (2019-09-23)
PBIO1969 (2019-09-24)
PBIO1968 (2019-09-25)
PBIO1967 (2019-09-25)
PBIO1980 (2019-09-25)
PBIO1997 (2019-10-01)
PBIO1996 (2019-10-01)
PBIO1995 (2019-10-02)
PBIO1994 (2019-10-04)
PBIO1993 (2019-10-06)
PBIO1992 (2019-10-16)
PBIO1991 (2019-10-21)
PBIO1990 (2019-10-23)
PBIO1989 (2019-10-24)
PBIO2003 (2019-10-24)
PBIO1988 (2019-11-03)
PBIO2002 (2019-11-03)
PBIO2011 (2019-12-09)
PBIO2012 (2019-12-16)
PBIO2018 (2020-01-08)
Klebsiella pneumoniae ST307 isolate (date)

Fig. S5. Single-nucleotide polymorphism (SNP) distribution of *K. pneumoniae* ST307 isolates. The horizontal axis shows the isolates sorted by isolation date. Top: Overview of SNP counts for intergenic SNPs and SNPs occurring in coding sequences (synonymous, missense and nonsense). Bottom: Detailed overview of SNP positions in the replicons based on the reference genome of PBIO1953 (Chr – chromosome; P1-5 – Plasmids 1-5). SNPs were filtered to retain those occurring in coding sequences (CDS) that are non-synonymous (i.e. missense/nonsense variants).

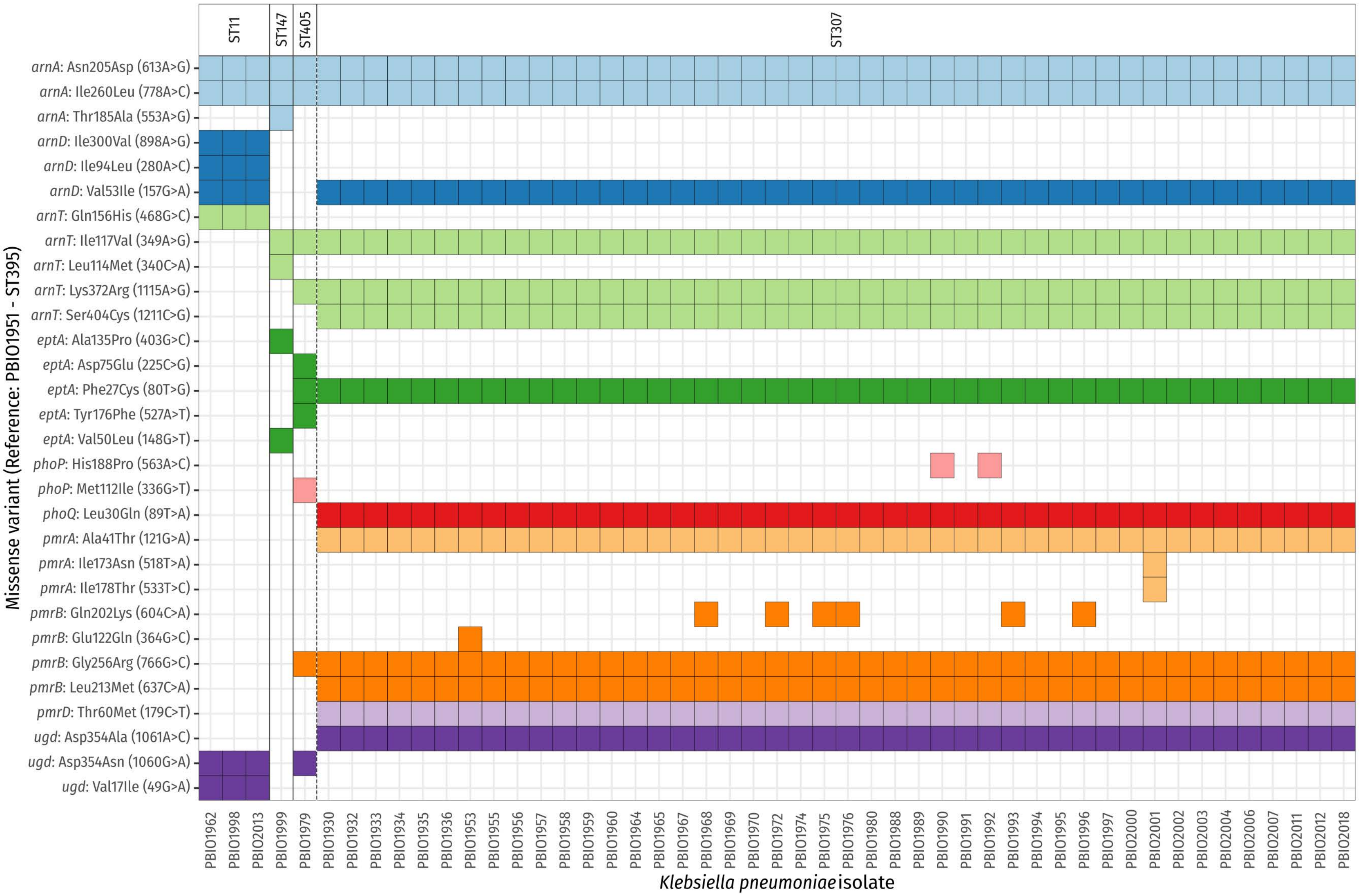

ST11
ST147
ST405
ST307
arnA: Asn205Asp (613A>G)
arnA: Ile260Leu (778A>C)
arnA: Thr185Ala (553A>G)
arnD: Ile300Val (898A>G)
arnD: Ile94Leu (280A>C)
arnD: Val53Ile (157G>A)
arnT: Gln156His (468G>C)
arnT: Ile117Val (349A>G)
arnT: Leu114Met (340C>A)
arnT: Lys372Arg (1115A>G)
arnT: Ser404Cys (1211C>G)
eptA: Ala135Pro (403G>C)
eptA: Asp75Glu (225C>G)
eptA: Phe27Cys (80T>G)
eptA: Tyr176Phe (527A>T)
eptA: Val50Leu (148G>T)
phoP: His188Pro (563A>C)
phoP: Met112Ile (336G>T)
phoQ: Leu30Gln (89T>A)
pmrA: Ala41Thr (121G>A)
pmrA: Ile173Asn (518T>A)
pmrA: Ile178Thr (533T>C)
pmrB: Gln202Lys (604C>A)
pmrB: Glu122Gln (364G>C)
pmrB: Gly256Arg (766G>C)
pmrB: Leu213Met (637C>A)
pmrD: Thr60Met (179C>T)
ugd: Asp354Ala (1061A>C)
ugd: Asp354Asn (1060G>A)
ugd: Val17Ile (49G>A)
Missense variant (Reference: PBIO1951 - ST395)
PBIO1962
PBIO1998
PBIO2013
PBIO1999
PBIO1979
PBIO1930
PBIO1932
PBIO1933
PBIO1934
PBIO1935
PBIO1936
PBIO1953
PBIO1955
PBIO1956
PBIO1957
PBIO1958
PBIO1959
PBIO1960
PBIO1964
PBIO1965
PBIO1967
PBIO1968
PBIO1969
PBIO1970
PBIO1972
PBIO1974
PBIO1975
PBIO1976
PBIO1980
PBIO1988
PBIO1989
PBIO1990
PBIO1991
PBIO1992
PBIO1993
PBIO1994
PBIO1995
PBIO1996
PBIO1997
PBIO2000
PBIO2001
PBIO2002
PBIO2003
PBIO2004
PBIO2006
PBIO2007
PBIO2011
PBIO2012
PBIO2018
Klebsiella pneumoniae isolate

Fig. S6. Missense variants in genes putatively related to colistin resistance. Raw reads of isolates were mapped against colistin-susceptible *K. pneumoniae* ST395 isolate PBIO1951. The vertical dashed line separates phenotypic colistin-susceptible (left) from colistin-resistant isolates (right). Filled squares (colored by gene) mark the presence of a missense variant (vertical axis) in the corresponding isolate (horizontal axis). Note that the two other Kp ST395 isolates (PBIO1961, PBIO2005) are not depicted as they did not show any of the listed variants.

A

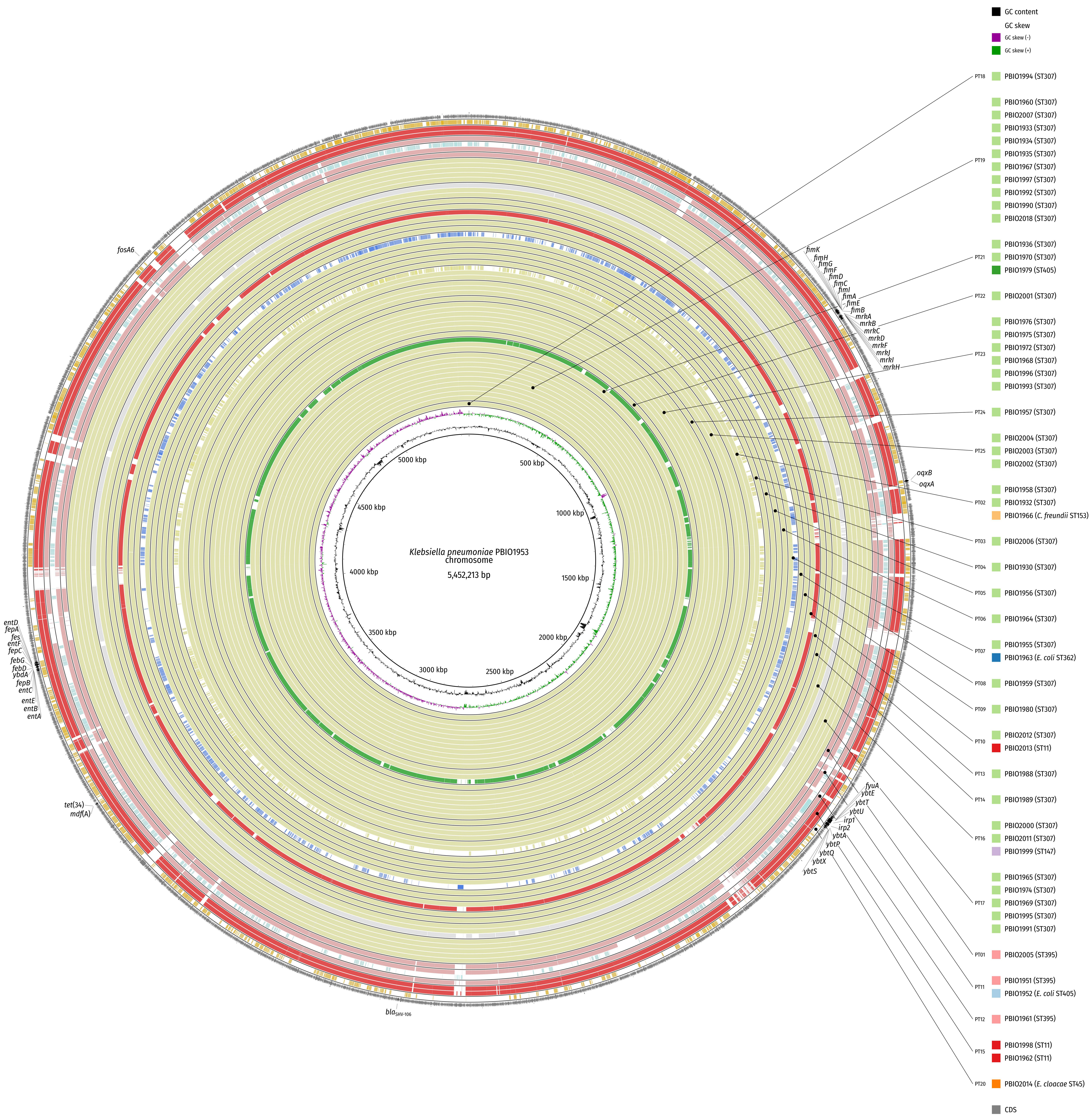
Klebsiella pneumoniae PBIO1953 chromosome
5,452,213 bp
500 kbp
1000 kbp
1500 kbp
2000 kbp
2500 kbp
3000 kbp
3500 kbp
4000 kbp
4500 kbp
5000 kbp
fosA6
oqxB
oqxA
tet(34)
mdf(A)
blaSHV-106
fimK
fimH
fimG
fimF
fimD
fimC
fimI
fimA
fimE
fimB
mrkA
mrkB
mrkC
mrkD
mrkF
mrkJ
mrkI
mrkH
entD
fepA
fes
entF
fepC
febG
febD
ybdA
fepB
entC
entE
entB
entA
fyuA
ybtE
ybtT
ybtU
irp1
irp2
ybtA
ybtP
ybtQ
ybtX
ybtS
GC content
GC skew
GC skew (-)
GC skew (+)
PT18 PBIO1994 (ST307)
PBIO1960 (ST307)
PBIO2007 (ST307)
PBIO1933 (ST307)
PBIO1934 (ST307)
PBIO1935 (ST307)
PT19 PBIO1967 (ST307)
PBIO1997 (ST307)
PBIO1992 (ST307)
PBIO1990 (ST307)
PBIO2018 (ST307)
PBIO1936 (ST307)
PBIO1970 (ST307)
PT21 PBIO1979 (ST405)
PT22 PBIO2001 (ST307)
PBIO1976 (ST307)
PBIO1975 (ST307)
PBIO1972 (ST307)
PT23 PBIO1968 (ST307)
PBIO1996 (ST307)
PBIO1993 (ST307)
PT24 PBIO1957 (ST307)
PBIO2004 (ST307)
PT25 PBIO2003 (ST307)
PBIO2002 (ST307)
PBIO1958 (ST307)
PT02 PBIO1932 (ST307)
PBIO1966 (C. freundii ST153)
PT03 PBIO2006 (ST307)
PT04 PBIO1930 (ST307)
PT05 PBIO1956 (ST307)
PT06 PBIO1964 (ST307)
PBIO1955 (ST307)
PT07 PBIO1963 (E. coli ST362)
PT08 PBIO1959 (ST307)
PT09 PBIO1980 (ST307)
PBIO2012 (ST307)
PT10 PBIO2013 (ST11)
PT13 PBIO1988 (ST307)
PT14 PBIO1989 (ST307)
PBIO2000 (ST307)
PT16 PBIO2011 (ST307)
PBIO1999 (ST147)
PBIO1965 (ST307)
PBIO1974 (ST307)
PT17 PBIO1969 (ST307)
PBIO1995 (ST307)
PBIO1991 (ST307)
PT01 PBIO2005 (ST395)
PBIO1951 (ST395)
PT11 PBIO1952 (E. coli ST405)
PT12 PBIO1961 (ST395)
PBIO1998 (ST11)
PT15 PBIO1962 (ST11)
PT20 PBIO2014 (E. cloacae ST45)
CDS

B

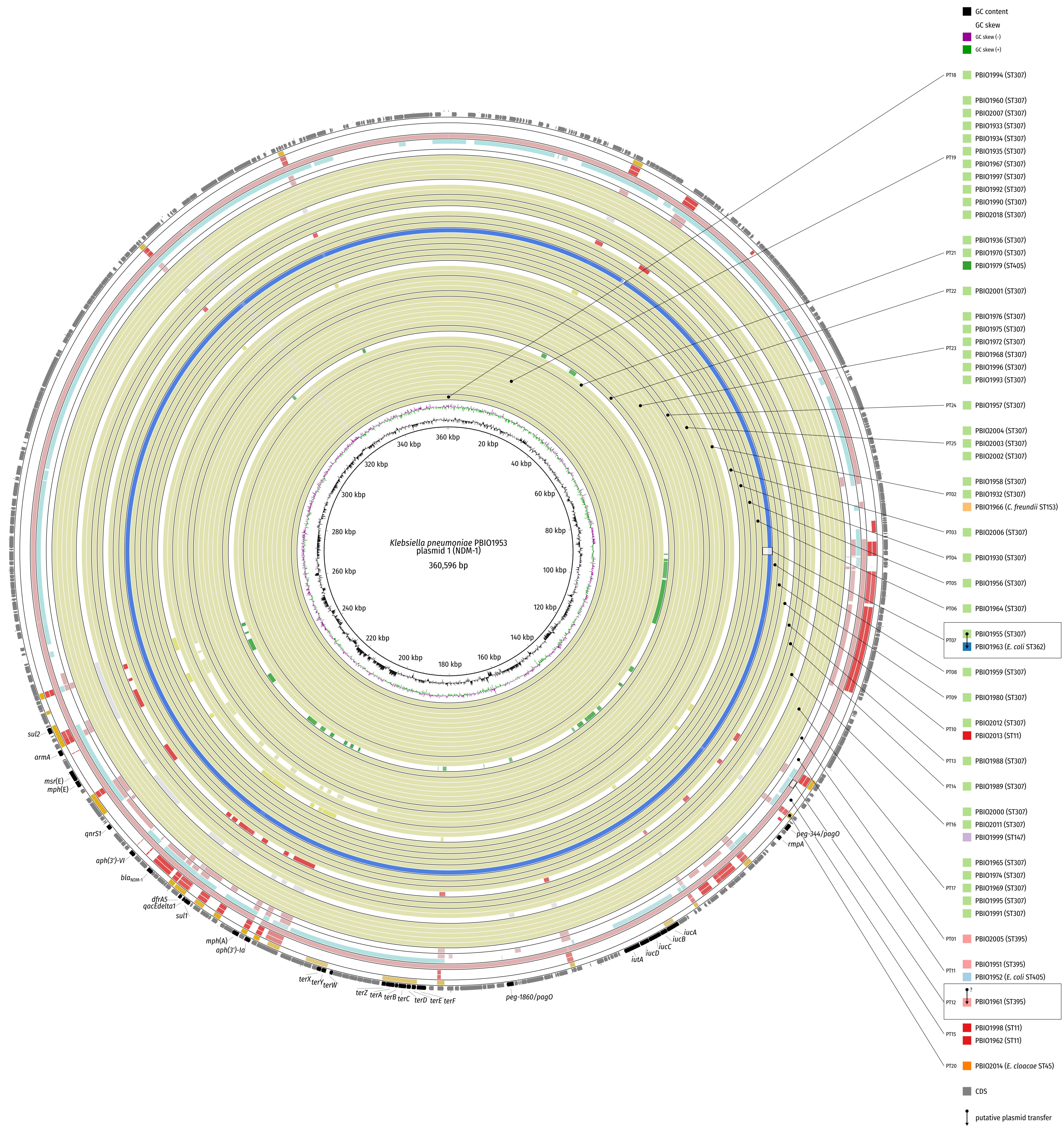
GC content
GC skew
GC skew (-)
GC skew (+)
PT18 PBIO1994 (ST307)
PBIO1960 (ST307)
PBIO2007 (ST307)
PBIO1933 (ST307)
PBIO1934 (ST307)
PBIO1935 (ST307)
PT19 PBIO1967 (ST307)
PBIO1997 (ST307)
PBIO1992 (ST307)
PBIO1990 (ST307)
PBIO2018 (ST307)
PBIO1936 (ST307)
PT21 PBIO1970 (ST307)
PBIO1979 (ST405)
PT22 PBIO2001 (ST307)
PBIO1976 (ST307)
PBIO1975 (ST307)
PT23 PBIO1972 (ST307)
PBIO1968 (ST307)
PBIO1996 (ST307)
PBIO1993 (ST307)
PT24 PBIO1957 (ST307)
PBIO2004 (ST307)
PT25 PBIO2003 (ST307)
PBIO2002 (ST307)
PBIO1958 (ST307)
PT02 PBIO1932 (ST307)
PBIO1966 (C. freundii ST153)
PT03 PBIO2006 (ST307)
PT04 PBIO1930 (ST307)
PT05 PBIO1956 (ST307)
PT06 PBIO1964 (ST307)
PBIO1955 (ST307)
PT07 PBIO1963 (E. coli ST362)
PT08 PBIO1959 (ST307)
PT09 PBIO1980 (ST307)
PBIO2012 (ST307)
PT10 PBIO2013 (ST11)
PT13 PBIO1988 (ST307)
PT14 PBIO1989 (ST307)
PBIO2000 (ST307)
PT16 PBIO2011 (ST307)
PBIO1999 (ST147)
PBIO1965 (ST307)
PBIO1974 (ST307)
PT17 PBIO1969 (ST307)
PBIO1995 (ST307)
PBIO1991 (ST307)
PT01 PBIO2005 (ST395)
PBIO1951 (ST395)
PT11 PBIO1952 (E. coli ST405)
?
PT12 PBIO1961 (ST395)
PBIO1998 (ST11)
PT15 PBIO1962 (ST11)
PT20 PBIO2014 (E. cloacae ST45)
CDS
putative plasmid transfer
Klebsiella pneumoniae PBIO1953
plasmid 1 (NDM-1)
360,596 bp
360 kbp
20 kbp
40 kbp
60 kbp
80 kbp
100 kbp
120 kbp
140 kbp
160 kbp
180 kbp
200 kbp
220 kbp
240 kbp
260 kbp
280 kbp
300 kbp
320 kbp
340 kbp
sul2
armA
msr(E)
mph(E)
qnrS1
aph(3')-VI
blaNDM-1
dfrA5
qacEdelta1
sul1
mph(A)
aph(3')-Ia
terX
terY
terW
terZ
terA
terB
terC
terD
terE
terF
peg-1860/pagO
iutA
iucD
iucC
iucB
iucA
peg-344/pagO
rmpA

C

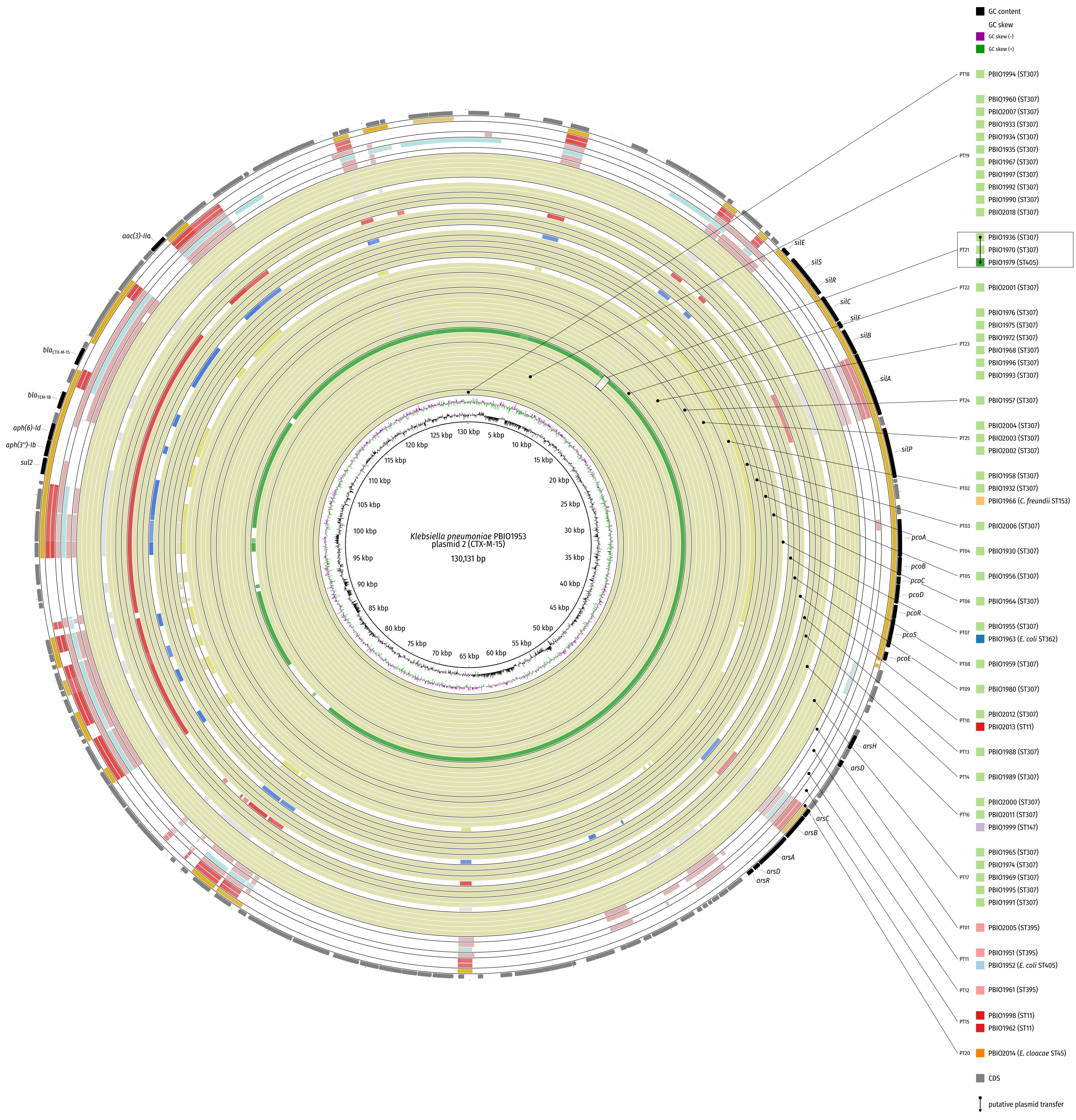
Klebsiella pneumoniae PBIO1953
plasmid 2 (CTX-M-15)
130,131 bp
5 kbp
10 kbp
15 kbp
20 kbp
25 kbp
30 kbp
35 kbp
40 kbp
45 kbp
50 kbp
55 kbp
60 kbp
65 kbp
70 kbp
75 kbp
80 kbp
85 kbp
90 kbp
95 kbp
100 kbp
105 kbp
110 kbp
115 kbp
120 kbp
125 kbp
130 kbp
aac(3)-IIa
blaCTX-M-15
blaTEM-1B
aph(6)-Id
aph(3'')-Ib
sul2
silE
silS
silR
silC
silF
silB
silA
silP
pcoA
pcoB
pcoC
pcoD
pcoR
pcoS
pcoE
arsH
arsD
arsC
arsB
arsA
arsD
arsR
GC content
GC skew
GC skew (-)
GC skew (+)
PT18
PBIO1994 (ST307)
PT19
PBIO1960 (ST307)
PBIO2007 (ST307)
PBIO1933 (ST307)
PBIO1934 (ST307)
PBIO1935 (ST307)
PBIO1967 (ST307)
PBIO1997 (ST307)
PBIO1992 (ST307)
PBIO1990 (ST307)
PBIO2018 (ST307)
PT21
PBIO1936 (ST307)
PBIO1970 (ST307)
PBIO1979 (ST405)
PT22
PBIO2001 (ST307)
PT23
PBIO1976 (ST307)
PBIO1975 (ST307)
PBIO1972 (ST307)
PBIO1968 (ST307)
PBIO1996 (ST307)
PBIO1993 (ST307)
PT24
PBIO1957 (ST307)
PT25
PBIO2004 (ST307)
PBIO2003 (ST307)
PBIO2002 (ST307)
PT02
PBIO1958 (ST307)
PBIO1932 (ST307)
PBIO1966 (C. freundii ST153)
PT03
PBIO2006 (ST307)
PT04
PBIO1930 (ST307)
PT05
PBIO1956 (ST307)
PT06
PBIO1964 (ST307)
PT07
PBIO1955 (ST307)
PBIO1963 (E. coli ST362)
PT08
PBIO1959 (ST307)
PT09
PBIO1980 (ST307)
PT10
PBIO2012 (ST307)
PBIO2013 (ST11)
PT13
PBIO1988 (ST307)
PT14
PBIO1989 (ST307)
PT16
PBIO2000 (ST307)
PBIO2011 (ST307)
PBIO1999 (ST147)
PT17
PBIO1965 (ST307)
PBIO1974 (ST307)
PBIO1969 (ST307)
PBIO1995 (ST307)
PBIO1991 (ST307)
PT01
PBIO2005 (ST395)
PT11
PBIO1951 (ST395)
PBIO1952 (E. coli ST405)
PT12
PBIO1961 (ST395)
PT15
PBIO1998 (ST11)
PBIO1962 (ST11)
PT20
PBIO2014 (E. cloacae ST45)
CDS
putative plasmid transfer

D

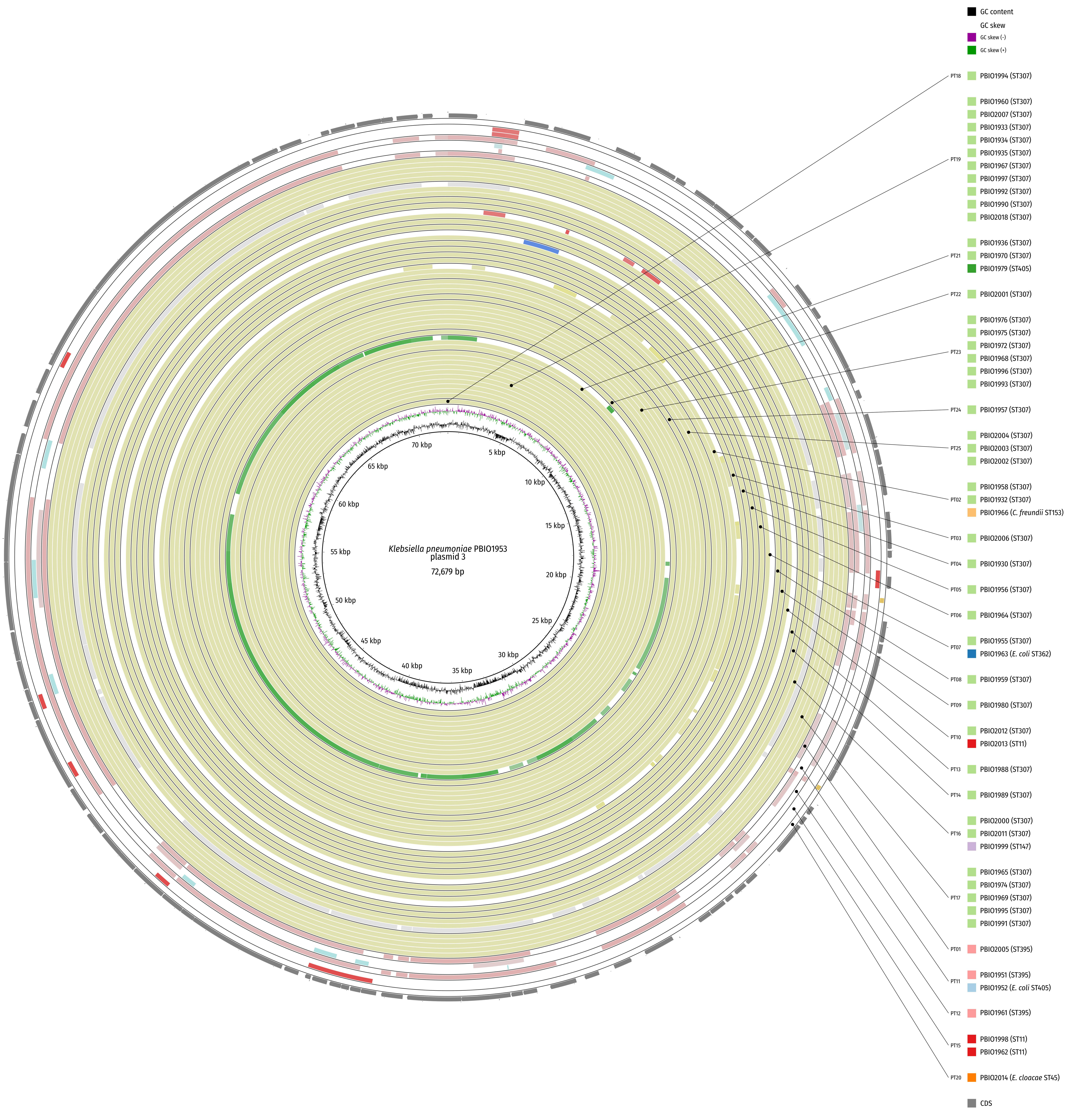
Klebsiella pneumoniae PBIO1953
plasmid 3
72,679 bp
5 kbp
10 kbp
15 kbp
20 kbp
25 kbp
30 kbp
35 kbp
40 kbp
45 kbp
50 kbp
55 kbp
60 kbp
65 kbp
70 kbp
GC content
GC skew
GC skew (-)
GC skew (+)
PT18
PBIO1994 (ST307)
PT19
PBIO1960 (ST307)
PBIO2007 (ST307)
PBIO1933 (ST307)
PBIO1934 (ST307)
PBIO1935 (ST307)
PBIO1967 (ST307)
PBIO1997 (ST307)
PBIO1992 (ST307)
PBIO1990 (ST307)
PBIO2018 (ST307)
PT21
PBIO1936 (ST307)
PBIO1970 (ST307)
PBIO1979 (ST405)
PT22
PBIO2001 (ST307)
PT23
PBIO1976 (ST307)
PBIO1975 (ST307)
PBIO1972 (ST307)
PBIO1968 (ST307)
PBIO1996 (ST307)
PBIO1993 (ST307)
PT24
PBIO1957 (ST307)
PT25
PBIO2004 (ST307)
PBIO2003 (ST307)
PBIO2002 (ST307)
PT02
PBIO1958 (ST307)
PBIO1932 (ST307)
PBIO1966 (C. freundii ST153)
PT03
PBIO2006 (ST307)
PT04
PBIO1930 (ST307)
PT05
PBIO1956 (ST307)
PT06
PBIO1964 (ST307)
PT07
PBIO1955 (ST307)
PBIO1963 (E. coli ST362)
PT08
PBIO1959 (ST307)
PT09
PBIO1980 (ST307)
PT10
PBIO2012 (ST307)
PBIO2013 (ST11)
PT13
PBIO1988 (ST307)
PT14
PBIO1989 (ST307)
PT16
PBIO2000 (ST307)
PBIO2011 (ST307)
PBIO1999 (ST147)
PT17
PBIO1965 (ST307)
PBIO1974 (ST307)
PBIO1969 (ST307)
PBIO1995 (ST307)
PBIO1991 (ST307)
PT01
PBIO2005 (ST395)
PT11
PBIO1951 (ST395)
PBIO1952 (E. coli ST405)
PT12
PBIO1961 (ST395)
PT15
PBIO1998 (ST11)
PBIO1962 (ST11)
PT20
PBIO2014 (E. cloacae ST45)
CDS

E

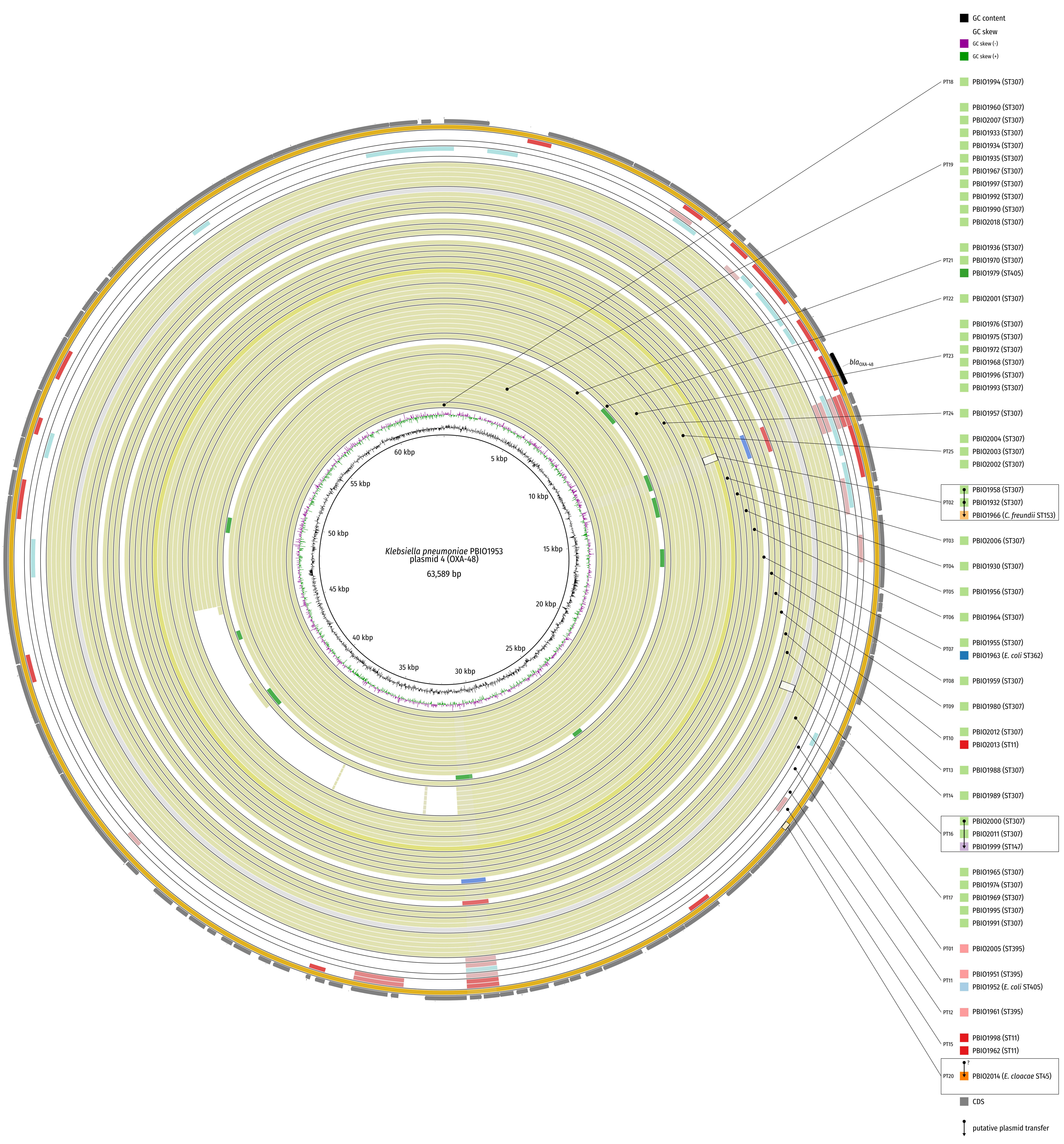

Klebsiella pneumoniae PBIO1953
plasmid 4 (OXA-48)
63,589 bp
5 kbp
10 kbp
15 kbp
20 kbp
25 kbp
30 kbp
35 kbp
40 kbp
45 kbp
50 kbp
55 kbp
60 kbp
blaOXA-48
GC content
GC skew
GC skew (-)
GC skew (+)
PT18 PBIO1994 (ST307)
PT19 PBIO1960 (ST307)
PBIO2007 (ST307)
PBIO1933 (ST307)
PBIO1934 (ST307)
PBIO1935 (ST307)
PBIO1967 (ST307)
PBIO1997 (ST307)
PBIO1992 (ST307)
PBIO1990 (ST307)
PBIO2018 (ST307)
PT21 PBIO1936 (ST307)
PBIO1970 (ST307)
PBIO1979 (ST405)
PT22 PBIO2001 (ST307)
PT23 PBIO1976 (ST307)
PBIO1975 (ST307)
PBIO1972 (ST307)
PBIO1968 (ST307)
PBIO1996 (ST307)
PBIO1993 (ST307)
PT24 PBIO1957 (ST307)
PT25 PBIO2004 (ST307)
PBIO2003 (ST307)
PBIO2002 (ST307)
PT02 PBIO1958 (ST307)
PBIO1932 (ST307)
PBIO1966 (C. freundii ST153)
PT03 PBIO2006 (ST307)
PT04 PBIO1930 (ST307)
PT05 PBIO1956 (ST307)
PT06 PBIO1964 (ST307)
PT07 PBIO1955 (ST307)
PBIO1963 (E. coli ST362)
PT08 PBIO1959 (ST307)
PT09 PBIO1980 (ST307)
PT10 PBIO2012 (ST307)
PBIO2013 (ST11)
PT13 PBIO1988 (ST307)
PT14 PBIO1989 (ST307)
PT16 PBIO2000 (ST307)
PBIO2011 (ST307)
PBIO1999 (ST147)
PT17 PBIO1965 (ST307)
PBIO1974 (ST307)
PBIO1969 (ST307)
PBIO1995 (ST307)
PBIO1991 (ST307)
PT01 PBIO2005 (ST395)
PT11 PBIO1951 (ST395)
PBIO1952 (E. coli ST405)
PT12 PBIO1961 (ST395)
PT15 PBIO1998 (ST11)
PBIO1962 (ST11)
?
PT20 PBIO2014 (E. cloacae ST45)
CDS
putative plasmid transfer

F

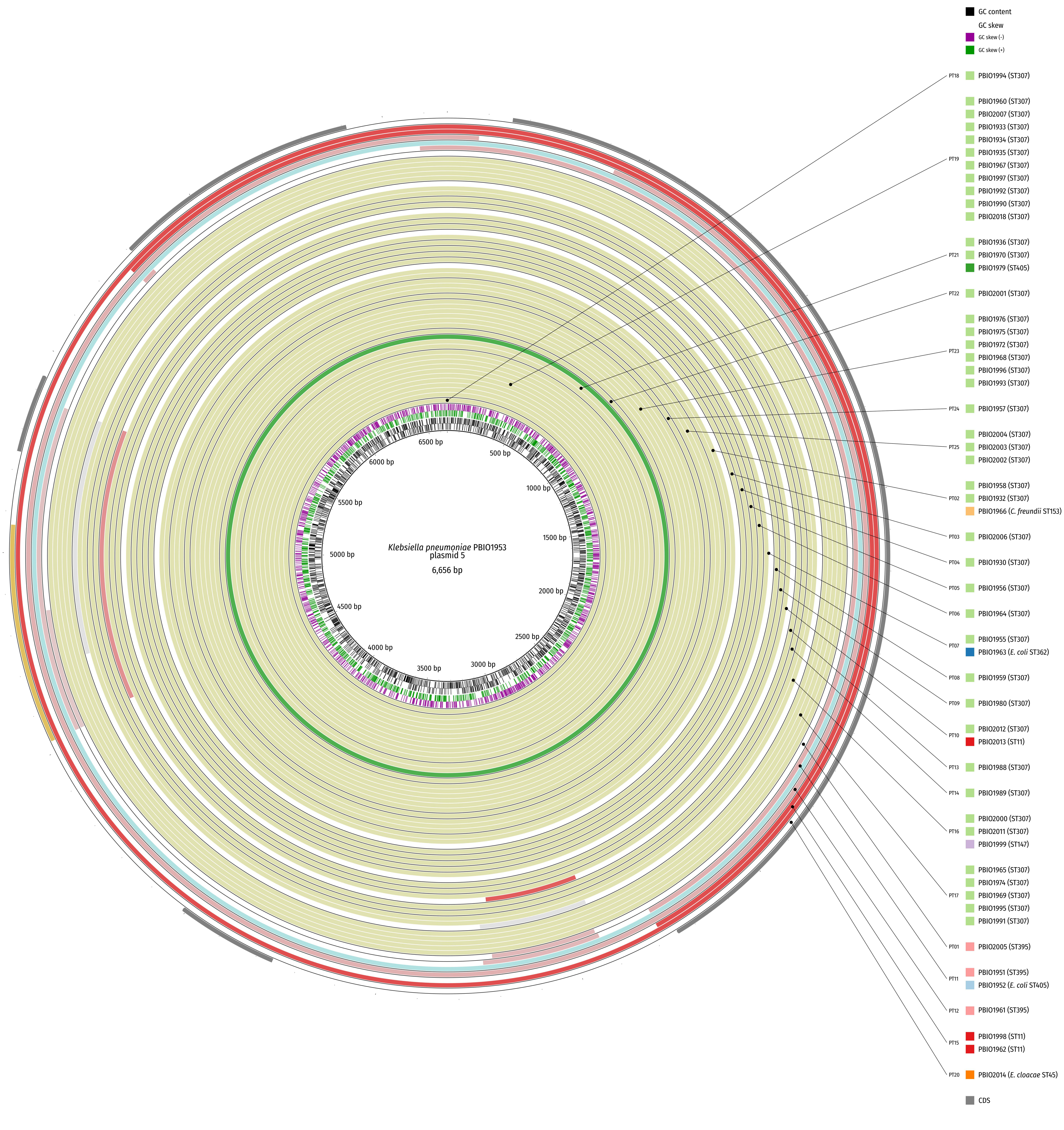

Klebsiella pneumoniae PBIO1953
plasmid 5
6,656 bp
500 bp
1000 bp
1500 bp
2000 bp
2500 bp
3000 bp
3500 bp
4000 bp
4500 bp
5000 bp
5500 bp
6000 bp
6500 bp
GC content
GC skew
GC skew (-)
GC skew (+)
PT18 PBIO1994 (ST307)
PBIO1960 (ST307)
PBIO2007 (ST307)
PBIO1933 (ST307)
PBIO1934 (ST307)
PBIO1935 (ST307)
PT19 PBIO1967 (ST307)
PBIO1997 (ST307)
PBIO1992 (ST307)
PBIO1990 (ST307)
PBIO2018 (ST307)
PBIO1936 (ST307)
PT21 PBIO1970 (ST307)
PBIO1979 (ST405)
PT22 PBIO2001 (ST307)
PBIO1976 (ST307)
PBIO1975 (ST307)
PBIO1972 (ST307)
PT23 PBIO1968 (ST307)
PBIO1996 (ST307)
PBIO1993 (ST307)
PT24 PBIO1957 (ST307)
PBIO2004 (ST307)
PT25 PBIO2003 (ST307)
PBIO2002 (ST307)
PBIO1958 (ST307)
PT02 PBIO1932 (ST307)
PBIO1966 (C. freundii ST153)
PT03 PBIO2006 (ST307)
PT04 PBIO1930 (ST307)
PT05 PBIO1956 (ST307)
PT06 PBIO1964 (ST307)
PBIO1955 (ST307)
PT07 PBIO1963 (E. coli ST362)
PT08 PBIO1959 (ST307)
PT09 PBIO1980 (ST307)
PBIO2012 (ST307)
PT10 PBIO2013 (ST11)
PT13 PBIO1988 (ST307)
PT14 PBIO1989 (ST307)
PBIO2000 (ST307)
PT16 PBIO2011 (ST307)
PBIO1999 (ST147)
PBIO1965 (ST307)
PBIO1974 (ST307)
PT17 PBIO1969 (ST307)
PBIO1995 (ST307)
PBIO1991 (ST307)
PT01 PBIO2005 (ST395)
PBIO1951 (ST395)
PT11 PBIO1952 (E. coli ST405)
PT12 PBIO1961 (ST395)
PBIO1998 (ST11)
PT15 PBIO1962 (ST11)
PT20 PBIO2014 (E. cloacae ST45)
CDS

Fig. S7. Comparison of the closed genome of PBIO1953 (PT18) with the other isolates. Shown are similarity search results of isolate contigs (Megablast, E-value 1e-10, low complexity filter turned off) against the PBIO1953 chromosome (A), plasmid 1 (B), plasmid 2 (C), plasmid 3 (D), plasmid 4 (E), and plasmid 5 (F) as colored arcs (respective sequence type). Isolates are grouped by patient (PT<number>) and the corresponding inner to outer concentric rings are connected to the legend from top to bottom. The outermost ring shows predicted coding sequences (CDS; gray) with antibiotic/metal resistance genes, virulence factors and biomarkers highlighted in black. Patients/isolates affected by a putative plasmid transfer are framed and the putative transfer is highlighted with an arrow.
